# The molecular biology of the olive fly comes of age

**DOI:** 10.1186/1471-2156-15-S2-S8

**Published:** 2014-12-01

**Authors:** Efthimia Sagri, Martin Reczko, Konstantina T Tsoumani, Maria-Eleni Gregoriou, Vaggelis Harokopos, Anna-Maria Mavridou, Spyros Tastsoglou, Konstantinos Athanasiadis, Jiannis Ragoussis, Kostas D Mathiopoulos

**Affiliations:** 1 Department of Biochemistry and Biotechnology, University of Thessaly, Larissa, Greece; 2 Institute of Molecular Biology and Genetics, Biomedical Sciences Research Centre "Alexander Fleming", Greece

**Keywords:** Tephritidae, Bactrocera oleae, sry-α, hid

## Abstract

**Background:**

Olive cultivation blends with the history of the Mediterranean countries since ancient times. Even today, activities around the olive tree constitute major engagements of several people in the countryside of both sides of the Mediterranean basin. The olive fly is, beyond doubt, the most destructive pest of cultivated olives. The female fly leaves its eggs in the olive fruit. Upon emergence, the larvae feed on the olive sap, thus destroying the fruit. If untreated, practically all olives get infected. The use of chemical insecticides constitutes the principal olive fly control approach. The Sterile Insect Technique (SIT), an environmentally friendly alternative control method, had been tried in pilot field applications in the 1970's, albeit with no practical success. This was mainly attributed to the low, non-antagonistic quality of the mixed-sex released insects. Many years of experience from successful SIT applications in related species, primarily the Mediterranean fruit fly, *Ceratitis capitata*, demonstrated that efficient SIT protocols require the availability of fundamental genetic and molecular information.

**Results:**

Among the primary systems whose understanding can contribute towards novel SIT approaches (or its recently developed alternative RIDL: Release of Insects carrying a Dominant Lethal) is the reproductive, since the ability to manipulate the reproductive system would directly affect the insect's fertility. In addition, the analysis of early embryonic promoters and apoptotic genes would provide tools that confer dominant early-embryonic lethality during mass-rearing. Here we report the identification of several genes involved in these systems through whole transcriptome analysis of female accessory glands (FAGs) and spermathecae, as well as male testes. Indeed, analysis of differentially expressed genes in these tissues revealed higher metabolic activity in testes than in FAGs/spermathecae. Furthermore, at least five olfactory-related genes were shown to be differentially expressed in the female and male reproductive systems analyzed. Finally, the expression profile of the embryonic *serendipity-α *locus and the pre-apoptotic *head involution defective *gene were analyzed during embryonic developmental stages.

**Conclusions:**

Several years of molecular studies on the olive fly can now be combined with new information from whole transcriptome analyses and lead to a deep understanding of the biology of this notorious insect pest. This is a prerequisite for the development of novel embryonic lethality female sexing strains for successful SIT efforts which, combined with improved mass-reared conditions, give new hope for efficient SIT applications for the olive fly.

## Background

When Athena, the goddess of peace and wisdom, offered an olive tree to the people of Attica to sway them into choosing her name for their city - and not that of her brother's Poseidon - neither she nor the people of Attica were aware of the 'worm' that could destroy the precious fruit of that tree. That was described much later in the 3^rd ^century AD, by the botanist Theophrastus who, in his works *"Enquiry into Plants" *and *"Causes of Plants" *[1], talked about the 'worm underneath the skin of the olive that destroys the fruit'. Indeed, the female olive fly (*Bactrocera oleae*, Rossi) lays her eggs in an olive fruit and the resulting larva feeds on the olive sap, opening channels inside it, thus destroying it. In this way, a female fly can damage more than 300 olives in her lifetime. Given the fact that during the summer and fall months about five generations of these flies are born, one can imagine the cumulative damage that can take place in an olive orchard. If untreated, practically every single olive will get infested. It is estimated that due to olive fly infestation olive oil production is reduced by more than 30% annually [2].

Control of these flies is traditionally based on cover or bait sprays with chemical insecticides. During the last 40-50 years, organophosphate insecticides have been extensively used against the olive fly, mainly dimethoate and fenthion. More recently, pyrethroids as well as the naturalyte spinosad have been added in the arsenal against the olive fly. The use of chemical pesticides, however, entails many known hazards. Among these are ecological disturbances, the development and spread of insecticide resistance, harmful toxicological effects on human health [3]. Many of these risks are apparent not only to scientists but also to growers and consumers who require a cleaner and safer environment as well as products of high quality. Alternative, environmentally friendly control methods against insect pests, such as the Sterile Insect Technique (SIT) have been experimented in the past with considerable success [4]. The SIT involves the mass production, sterilization and subsequent release of the sterilized insects [5]. The sterilized males will mate with wild females, whose unfertilized eggs will never hatch, thus reducing the numbers of the following generation. In theory, if continued releases are performed over several consecutive generations, the population will progressively be reduced and, eventually, a total eradication could occur.

Given the substantial economic burden of the olive fly in olive producing countries and the concerns raised about the heavy use of insecticides to control the flies, the SIT was proposed [6] and implemented in two pilot efforts. In the early 1970s, about 150,000 laboratory-reared male and female flies were sterilized by gamma-irradiation and subsequently released in the environment [7]. Although initially the releases seemed to contribute to low infestation levels, by the end of the season olives were as highly infested as in the two nearby control plantations. The sterilized flies were proven ineffective to reduce infestation. Similar results were obtained in a second pilot SIT effort that took place in the late '70s in a small Greek island. These unsuccessful pilot experiments led to funding suspension and the eventual abandonment of the program [8-10]. Apart from the high cost and labor-intensive rearing of the olive fly, extensive research that followed these first pilot efforts revealed several key issues of olive fly biology that should have been sorted out before a successful SIT could be implemented. The first issue regarded assortative mating of the released and wild populations. Laboratory-reared flies mated several hours before scotophase whereas wild flies mated at the end of the photophase [11]. Apparently, mass-laboratory rearing caused substantial alterations in the genetic makeup of the flies due to selective pressures in the artificial laboratory environment [12,13]. The second issue regarded the quality of the radiation-sterilized mass-reared flies. Radiation did not leave the vigor of the flies unaffected [14]. Another factor that probably exacerbated the low fitness of the laboratory reared flies was the use of antibiotics in the flies' diet that destroyed the endosymbiotic bacteria that are now known to play a very important role in the organism's fitness [15-19]. Finally, but equally importantly, extensive stinging of the olive fruits from the released females led to further fungal infestation [7].

Since those early years, several molecular and genetic studies have changed *B. oleae*'s research landscape. First, the development of microsatellite markers [20] and the analysis of the mitochondrial genome [21] have offered tools for a fairly detailed analysis of population structure and dynamics in the Mediterranean basin [22-26]. Second, cytogenetic analysis, including *in situ *hybridization of several molecular markers, established the details of the chromosomal complement [27-31]. Third, isolation and characterization of various genes has shed light on important processes such as insecticide resistance [32-35], female germline differentiation and morphogenesis of epidermal cells [36], enzyme catalytic mechanisms [37], sex-determining cascades [38,39]. Fourth, an initial assessment of the genome of the olive fly was gained by an accurate estimate of its size [40] and the characterization and analysis of centromeric repeats [41] and several EST loci [42]. This was followed by a whole transcriptome analysis with 454 pyrosequencing [43]. Fifth, *B. oleae *was successfully transformed with the use of a *Minos*-based transposon [44]. Transformation efforts recently led to the development of *piggyBac*-based conditional female-lethal olive fly strains that provide highly penetrant female specific lethality, dominant fluorescent marking and genetic sterility [45]. Sixth, *B. oleae *was recently trans-infected with a cherry fly *Wolbachia *strain and shown to induce complete cytoplasmic incompatibility in the fly [46]. Finally, the experience gained during the first two pilot SIT efforts and the relevant research that followed, underlined a few key requirements for the maintenance of high quality and well-fit mass-reared olive flies (reviewed in [47]). Among them were changes in larval and adult diets (eg removal of antibiotics) that would preserve the endosymbiotic flora (that is now known to improve fitness) and occasional enrichments of the long-term laboratory colonies with wild individuals (that provide natural vigor). These achievements have renewed the interest in using SIT for olive fly control. In fact, there is a large international effort led by the Joint Division of the Food and Agricultural Organization and the International Atomic Energy Agency (FAO/IAEA) to develop a vigorous laboratory olive fly strain that could be used in such new SIT efforts.

Further scientific and technological developments, in addition to successful SIT applications in other insects, point to the direction olive fly research could go. Indeed, SIT has proven particularly effective in the medfly, the prototype Tephritid species where most genetic and molecular tools have been developed. One of the most active medfly research areas in recent years has been the development of the RIDL technology. RIDL (Release of Insects carrying a Dominant Lethal; [48,49]) is a variant of the conventional SIT, in which sterilization of the released insects is induced not by irradiation but by homozygocity for a dominant lethal gene. Mating with wild individuals results in offspring that are heterozygous for the lethal gene leading to the death of all progeny [50,51]. This dominant lethal gene can be placed under the control of an inducible early embryonic female promoter [51,52] that could achieve genetic sexing at a very early developmental stage. In this way, both genetic sexing and sterilization can be accomplished by the same construct. One other active research area regards the analysis of biological systems with relevance to SIT. Of particular interest are those that regard reproduction and olfaction. The first one is involved in successful mating and egg development, while the second in food and mate localization. A possible manipulation of either or both of these systems would severely affect the destructive ability of the flies. In that sense, transgenic flies could be developed in which genes regulating food and mate recognition or fertility are knocked-down, over-expressed or mis-expressed (depending on the case). Such flies would be safer and more efficient to be released in control programs in an SIT context.

The falling prices of next generation sequencing make it now possible to sequence the entire transcriptome of non-model organisms under different settings and identify differentially expressed genes relevant to the chosen conditions. Subsequently, these genes can be manipulated *in vitro *and re-introduced into the genome of the organism through well-established transgenic technologies. In a first attempt to explore the relevant-to-SIT transcriptome of the olive fly, we present differences observed in female and male reproductive systems and we examine the differential expression of olfactory genes in the same tissues. Finally, we assess the developmental expression of two of the most commonly used early embryonic genes.

## Results and discussion

### 1. Sequencing and annotation

#### 1.1. Solid ABI sequencing and reads assembly

In order to explore differentially expressed genes in the transcriptome of reproductive organs of the olive fly that could be useful in SIT development, the entire transcriptomes from female accessory glands and spermathecae were compared to male testes. For transcriptome assembly, the sequences from these two libraries (FEMALE and MALE) were combined with two more obtained from heads of spinosad-sensitive (LAB) and spinosad-resistant (SPIN) olive flies [53]. Paired-end sequencing with 35nt and 50nt read sizes was performed for each library and a total of 122,623,894 read pairs was obtained. All reads of the libraries were pooled to obtain a reference transcriptome assembly using SOAPdenovo assembler [54].

#### 1.2. Sequence annotation

Annotation of the assembled sequences was obtained by aligning the 69,359 assembled *B. oleae *sequences against the NCBI non-redundant (Nr) protein database using blastx and collecting the annotations with the BLAST2GO tool [55]. Using an E-value threshold of ≤1e^-6^, 20207 (29.13 %) of the contigs were aligned. Of the 69,359 contigs, 23,042 (33.22%) have almost exact hits in the *B. oleae *transcriptome of Pavlidi et al [43] (E-value ≤1e^-6^).

### 2. Female vs male differential expression

The Cuffdiff [56] tool was used in order to reveal the differentially expressed genes between the reproductive systems of female and male flies, a stringent cutoff (p value adjusted for multiple testing, called q value <0.05) was used. This resulted in 1568 differentially expressed transcripts in the FEMALE vs. MALE comparison. Three hundred and thirty of these transcripts were up-regulated in FEMALE, while 1238 were up-regulated in MALE *B. oleae *flies. The top 40 up-regulated genes in each category are listed in Table S1. The entire lists of all significantly (q<0.05) up-regulated genes in FEMALE and MALE are given in Tables S3 and S4, respectively.

**Figure 1:**
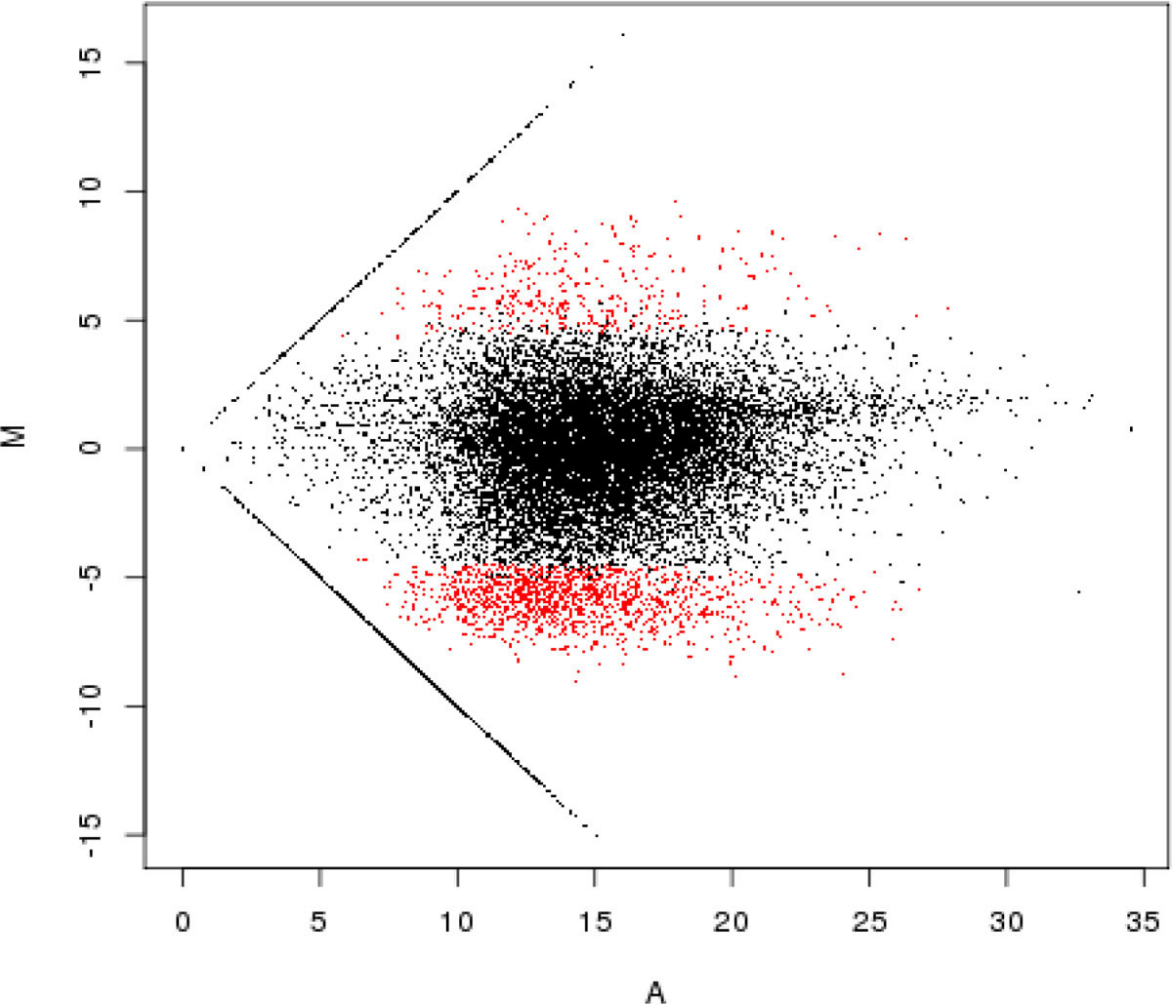
**M-A plot of gene expression for female and male flies**. Genes up-regulated in females have positive M values. Significantly differentially expressed genes (q-value < 0.05) are shown in red.

An M-A plot was constructed for comparison of the genes for FEMALE vs MALE flies with q value < 0.05. In Figure 1 the de-regulated genes are depicted in red.

**Figure 2:**
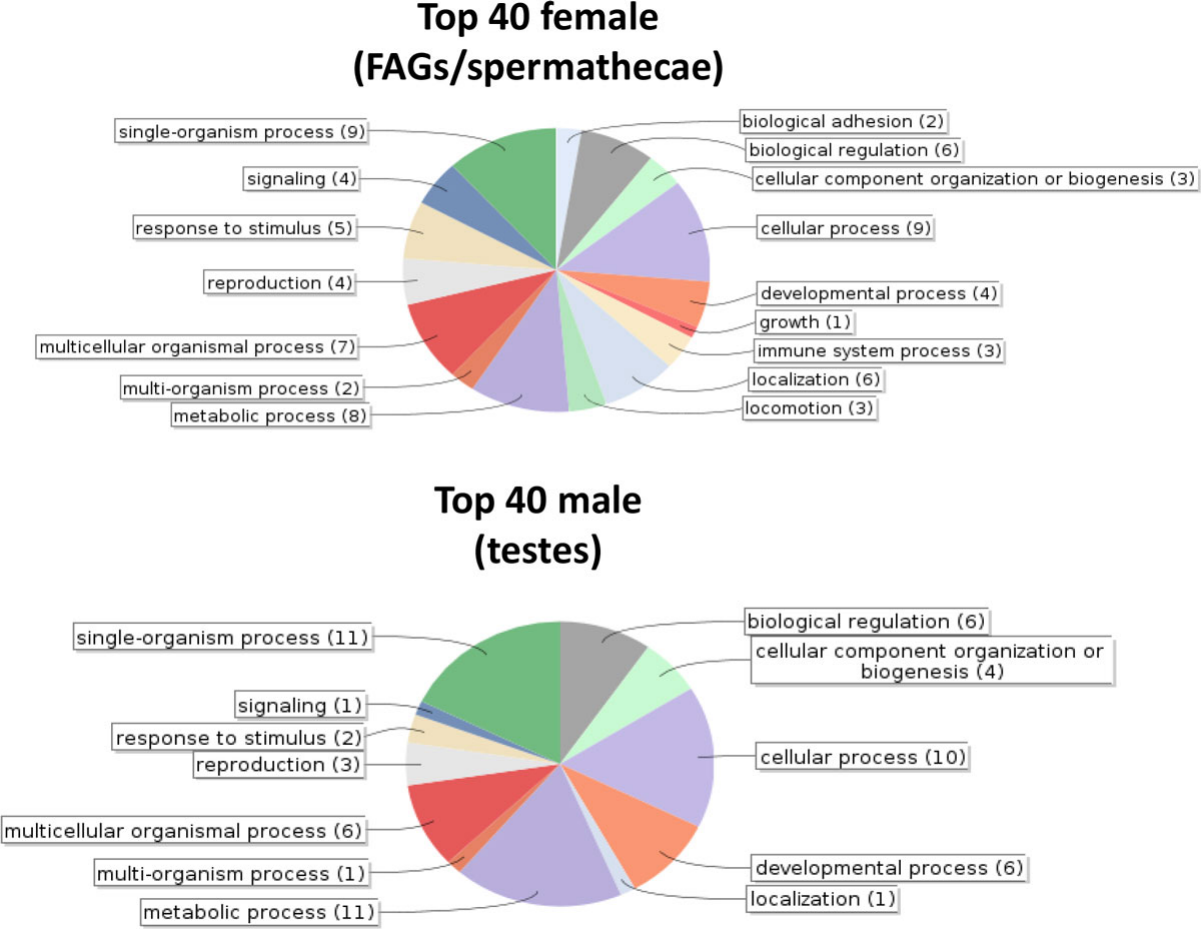
**GO Term associations for the top 40 genes expressed in the female and male tissues**. Associations were identified with BLAST2GO, using terms at the second level of the GO hierarchy.

Functional annotation was made for the assembled sequences of the significantly differentially expressed female- and male- specific genes mentioned in Table S1, based on gene ontology (GO) categorization obtained using BLAST2GO. The FEMALE and MALE GO analysis performed for biological process of the top 40 female and male expressed genes is shown in Figure 2. In general, more GO terms appear in female tissues than in male (16 vs 12), a point that holds even in deeper GO-term analysis. This can be attributed to the fact that the FEMALE library was comprised of both FAGs and spermathecae, while the MALE from testes only. Furthermore, there were more male- than female-specific genes involved in metabolism and development, a fact that can be attributed to sperm activity in the MALE tissue. Finally, the presence of three immune system process genes in the female list should be noted. In fact, increased levels of immune response genes have been found in transcriptome analyses of insect female reproductive systems, particularly after mating [57,58]. Upregulation of these genes may assist females to combat pathogens introduced during copulation. Alternatively, it could be a result of female's perception of sperm as non-self molecules.

**Figure 3:**
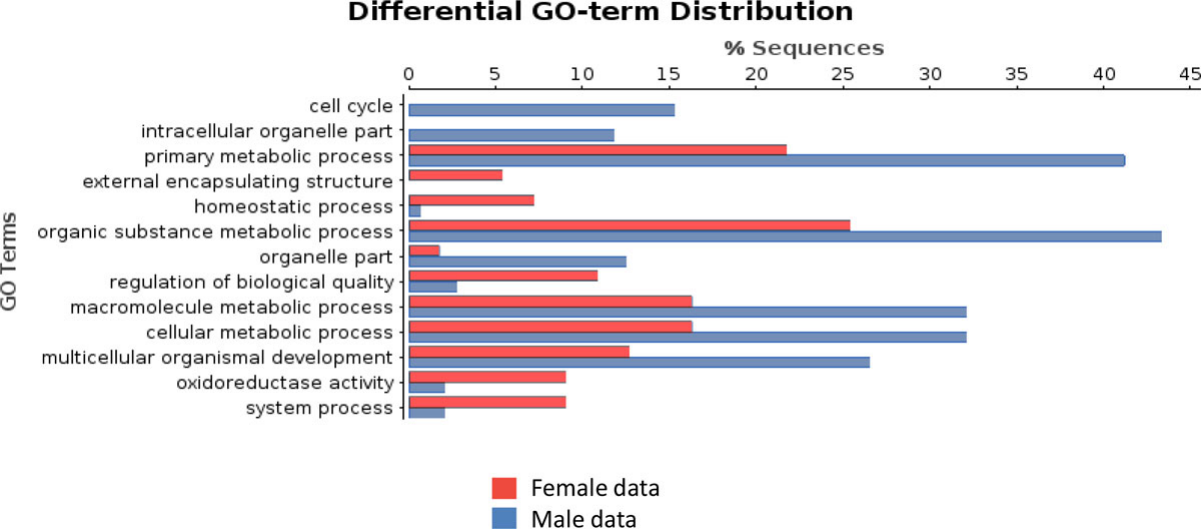
**GO-terms (GO-Slim) differentially distributed between male and female transcriptomes**. Contigs expressed only in female tissue are used as the test set (red bars) and contigs expressed only in male tissues as the reference set (blue bars).

A more direct comparison between FEMALE-only and MALE-only GO-term distribution is shown in Figure 3. Interestingly, numbers of GO-terms for biological process appear different in the two datasets, suggesting a different complexity of the studied female and male reproductive tissues. In most terms, there are more male- than female-specific transcripts that are differentially expressed. Many of these terms (cell cycle, intracellular organelle part, primary metabolic process, organic substance metabolic process, macromolecule metabolic process, cellular metabolic process, multicellular organismal development) refer to higher metabolic processes. This could be attributed to higher metabolic and cellular activity that takes place in the testes before mating.

### 3. Genes that might be implicated in sexual differentiation in *B. oleae*

**Figure 4:**
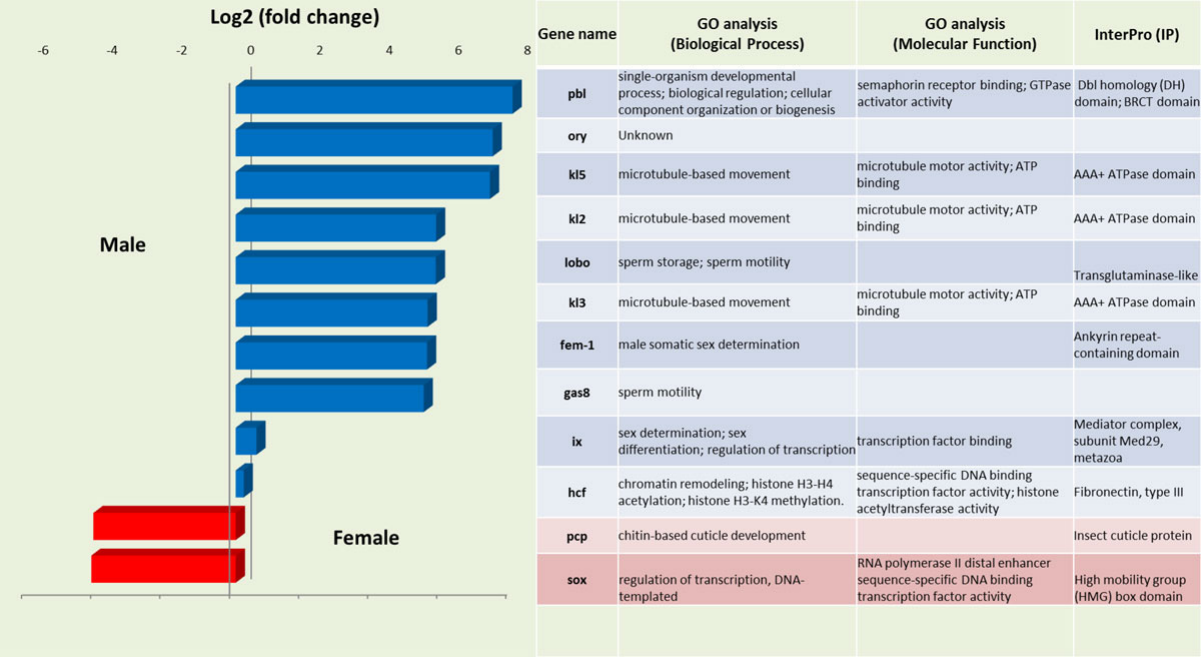
**Functional annotation of differentially expressed sex-differentiation genes**. In the left part of the figure, the gene expression levels of the differentially expressed sex-differentiation genes (Log2, fold change) are shown, as resulted from the RNA-seq analysis. The up-regulated genes in males are depicted in blue bars and the up-regulated genes in females in red bars. At the right part of the figure, the Gene Ontology (GO) classification of the same genes for the ontologies: Biological Process (BP), Molecular Function (MF), and Interpro (IP) protein domains is listed.

In order to validate the differential expression of various genes observed after the RNAseq analysis of reproductive tissues of female and male olive flies, further functional analysis was performed for twelve genes that were differentially expressed in female accessory glands and spermathecae, on one hand, and male testes, on the other (Figure 4). These genes were selected on the basis of known involvement in sexual differentiation in other insects. Seven of them were selected from the 1238 significantly up-regulated in MALE (Table S4): *kl2 (male fertility factor kl2), kl3 *(*male fertility factor kl3*), *kl5 *(*male fertility factor kl5*), *ory *(*occludin-related Y protein*), *fem-1 *(*sex-determining protein fem-1*), *gas8 *(*growth arrest specific protein 8*) and *lobo *(*lost boys*). Three more genes that were up-regulated in MALE [*ix *(*intersex*), *pbl *(*pebble*) and *hcf *(*host cell factor C1*)] and two that were up-regulated in FEMALE [*sox and pcp *(*pupal cuticle protein 78E*)], albeit with lower statistical power (i.e., q>0.05) were also selected for further validation.

#### 3.1. Drosophila Y-linked genes *kl3, kl5 *and *ory*

**Figure 5:**
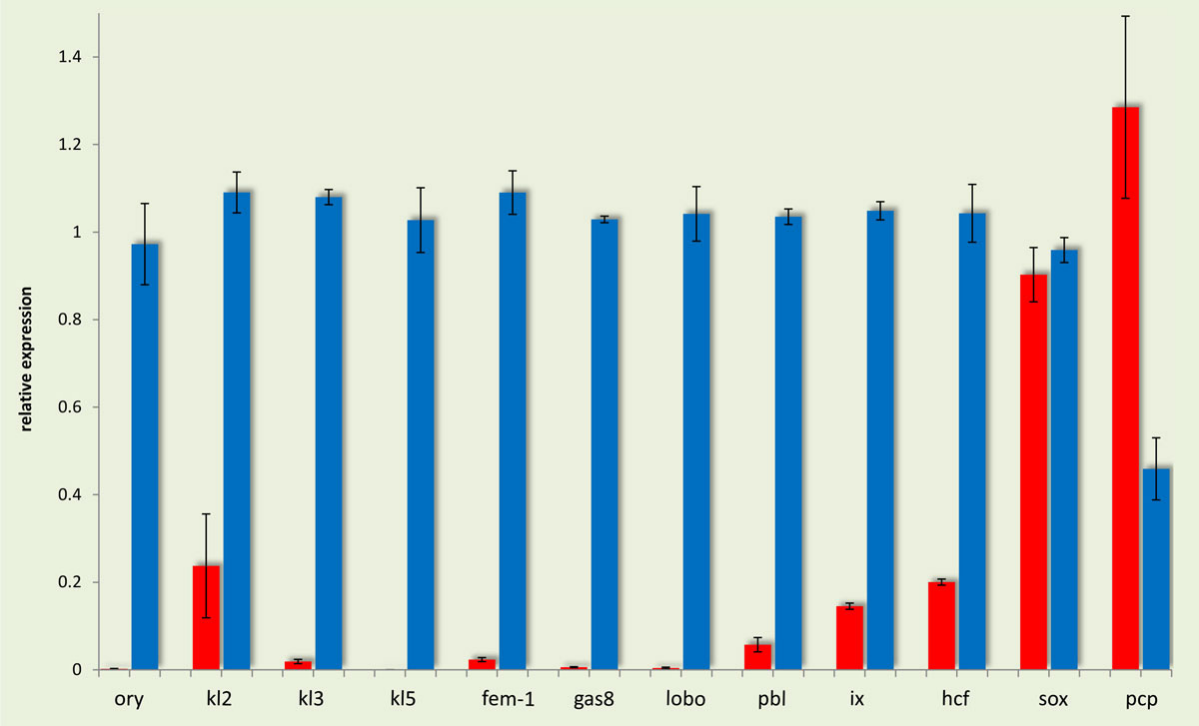
**Validation profiles of differentially expressed sex-differentiation genes**. Differentially expressed sex-differentiation genes of Figure 4 were further validated by qRT-PCR. Expression in male testes is depicted in blue color columns and expression in female accessory glands and spermathecae in red. Standard error of the mean of the two biological replicates is shown in bars. In all genes, except *sox *and *pcp*, expression in FEMALE and MALE was significantly different, as determined by t-test (p < 0.05).

Quantitative RT-PCR confirmed the elevated expression of *kl2, kl3, kl5 *and *ory *in male testes of the olive fly (Figure 5). In *Drosophila melanogaster, kl3 *and *kl5 *(along with *kl2*) are known Y-linked fertility factors. The lack of *kl3 *or *kl5 *causes the loss of the outer arm of the sperm tail axoneme [59], a structure known to contain the molecular motor protein dynein in other organisms [60]. Indeed, Goldstein et al. showed in 1982 that sperm from mutant *kl3^- ^*and *kl5^- ^*males lack three discrete high molecular weight proteins with mobility similar to dynein heavy chains of *Chlamydomonas reinhardtii *and proposed that these fertility factors are the structural genes of three different dynein heavy chain proteins [61]. In 1993, Gepner and Hays sequenced part of *kl5 *and showed that it encodes an axonemal β-dynein heavy chain that is expressed in the testes [62].

*ory *is also Y-linked in *D. melanogaster*, although details on this gene are scarce. *kl3, kl5 *and *ory *are Y-linked in 12 different sequenced Drosophila genomes [63]. In Drosophila, the closest paralogs of *kl2, kl3*, and *kl5 *are autosomal and not X-linked, suggesting that the evolution of the Drosophila Y chromosome has been driven by an accumulation of male-related genes arising *de novo *from the autosomes [64]. While the most likely function of the three genes in the olive fly might be similar to that of Drosophila, we have no indication with regard to their chromosomal localization in the olive fly. Such information could shed some light to the evolutionary origin of the olive fly's Y chromosome.

#### 3.2. Spermatogenesis and sperm motility genes

One spermatogenesis and two sperm motility genes were shown to be differentially over-expressed in male olive fly tissues both in the transcriptome analysis and after q-RT PCR (Figure 4 and 5). The first locus, ***sex-determining protein fem-1 (fem-1)***, encodes an essential spermatogenesis product in *Caenorhabditis elegans*. Three *fem *genes, *fem-1, fem-2*, and *fem-3*, have been shown to be essential for male development [65]. Loss-of-function mutations in any one of the *fem *genes prevent all aspects of male development and transform the animals that are genetically males into females [66,67]. The predicted product of the *fem-1 *gene is an intracellular protein that contains ankyrin repeats, which in many other proteins mediate specific protein-protein interaction [67]. In *D. melanogaster*, a *fem-1 *homolog with similar structure has been found [68]. The second locus, ***growth arrest-specific protein 8 (Gas8) ***is a microtubule-binding protein localized to regions of dynein regulation in mammalian cells. In mouse, Gas8 is predominantly a testicular protein, whose expression is developmentally regulated during puberty and spermatogenesis. In humans, it is absent in infertile males who lack the ability to generate gametes [69]. Gas8 has not been studied in insects. Finally, ***lost boys (lobo)***, has been shown to affect sperm entry movement into the female seminal receptacle and does not affect sperm exit movement from the seminal vesicle of *D. melanogaster *[70]. Given a similar function of these two loci in the olive fly, over-expression in male testes is expected.

#### 3.3. Sex determination genes

In *D. melanogaster, **intersex (ix) ***controls somatic sexual differentiation only in females, acting near the end of the sex determination hierarchy. Its product does not have a known DNA-binding domain and, therefore, it is thought to act as a transcriptional co-factor for the female variant of Doublesex protein (DSX^F^), a key gene of the sexual determination cascade in *D. melanogaster *[71]. Minimal differences were observed in *ix *expression between the two sexes of the olive flies.

Transcriptome analysis also showed a four-fold over-expression of ***sox ***in female tissues, a result that was not confirmed after validation. The *sox *gene family is a group of related transcription factors that play critical roles in embryonic development. This family was originally identified in mammals based on sequence similarity to SRY, the sex-determining region Y chromosome [72]. In the honeybee, as SOX proteins play key roles in gonad differentiation, the SoxE group orthologues were up-regulated in the drone testes [73]. In Drosophila SoxN is a new group B Sox gene expressed in the developing CNS and is one of the earliest transcription factors to be expressed in a pan-neuroectodermal manner [74].

#### 3.4. Other genes

The ***Pebble (pbl) ***gene belongs to a family of GTP exchange factors that are essential for the construction of a contractile ring and the initiation of cytokinesis during the embryonic division cycles of the somatic cells in *D. melanogaster *[75,76]. Its role in spermatogenesis has not been elucidated yet. Expression of *pbl *in *D. melanogaster *testes is low [68]. On the other hand, expression in olive fly testes was found elevated in comparison to its expression in female accessory glands/spermathecae (Figure 4 and 5).

***Host cell factor C1 (Hcf) ***is involved in a wide variety of cellular functions, including regulation of transcription, cytokinesis, cell cycle progression and chromatin remodeling [77]. The protein is essential for cellular viability and demonstrates similar activity among a broad range of species. A single *hcf *homolog is also present in *Drosophila *(called dHCF) and is expressed in all tissues, although at relatively low levels [68]. The transcriptome analysis in the olive fly tissues showed a ~0,2-fold higher expression in the male tissues. This result was confirmed after qRT-PCR in the same tissues, where higher levels of expression in testes were observed in comparison with female accessory glands/spermathecae (Figure 4 and 5).

Quantitation by RT-PCR confirmed the over-expression of ***pupal cuticle protein (pcp) ***in female accessory glands/spermathecae as compared to male testes. Cuticle proteins, along with chitin, are the two components of insect cuticle. The cuticular proteins seem to be specific to the type of cuticle that occurs at stages of the insect development. Flexible proteins are found in the flexible cuticle of larva and pupa, but can also be found in the soft endocuticle of adult insects [78].

Female insects require the steroid hormone 20-hydroxyecdysone (20E) in order to activate vitellogenesis, a process required for egg development. In *Anopheles gambiae *mosquitoes, large amounts of 20E are produced and stored in male accessory glands and subsequently delivered to female mosquitoes during mating [79]. Pupal cuticle proteins, on the other hand, are known to accumulate in response to a pulse of 20E [80]. However, given that FAGs/spermathecae collected were from unmated females, we cannot offer a plausible explanation for the over-expression of *pcp*s.

### 4. Validation of olfactory gene differential expression

Insects possess very sensitive chemosensory systems that can detect and discriminate among a diverse array of odors. These systems play a crucial role in insect survival and reproductive success, mediating responses to food detection, mating and oviposition. Odor recognition is a coordinated process requiring the combined specificities contributed by odorant-binding proteins (OBPs) and chemosensory proteins (CSPs) as well as odorant receptors (ORs) (Reviewed in [81]). Insect odorant-binding proteins (OBPs) are soluble proteins surrounding the extracellular lymph of olfactory neurons [82]. OBPs are capable of binding and solubilizing small hydrophobic molecules from the environment and therefore transport them to the underlying ORs, which are expressed on peripheral olfactory receptor neurons. Insect ORs are either ionotropic receptors (IRs) or seven-transmembrane proteins (ORs) with an inverse topology compared to GPCRs, that form heterodimers of a ligand-binding OR and an ubiquitous highly conserved co-receptor named Orco [83]. These complexes are suggested to constitute ligand-gated nonselective cation channels triggering the olfactory signaling [81].

While OR expression in olfactory tissues is obvious and well-established, the distribution of ORs beyond the olfactory system has also been documented in different mammalian species [84-86], suggesting that ORs may play an important role in the ectopic expression of non-chemosensory tissues. Interestingly, OR expression has been documented in human and mouse germ cells [87-91] and recently in mosquitoes [92]. Similarly, other non-olfactory functions have been reported for OBP-like proteins including the B proteins of *Tenebrio molitor *accessory glands [93], the male specific serum proteins of *Ceratitis capitata *[94], and the heme-binding protein of *Rhodnius prolixus *[95]. These demonstrate that OBPs are not restricted to olfaction and are likely to be involved in broader physiological functions, suggesting that their roles may be restricted to general carrier capabilities with broad specificity for lipophilic compounds [96].

**Figure 6:**
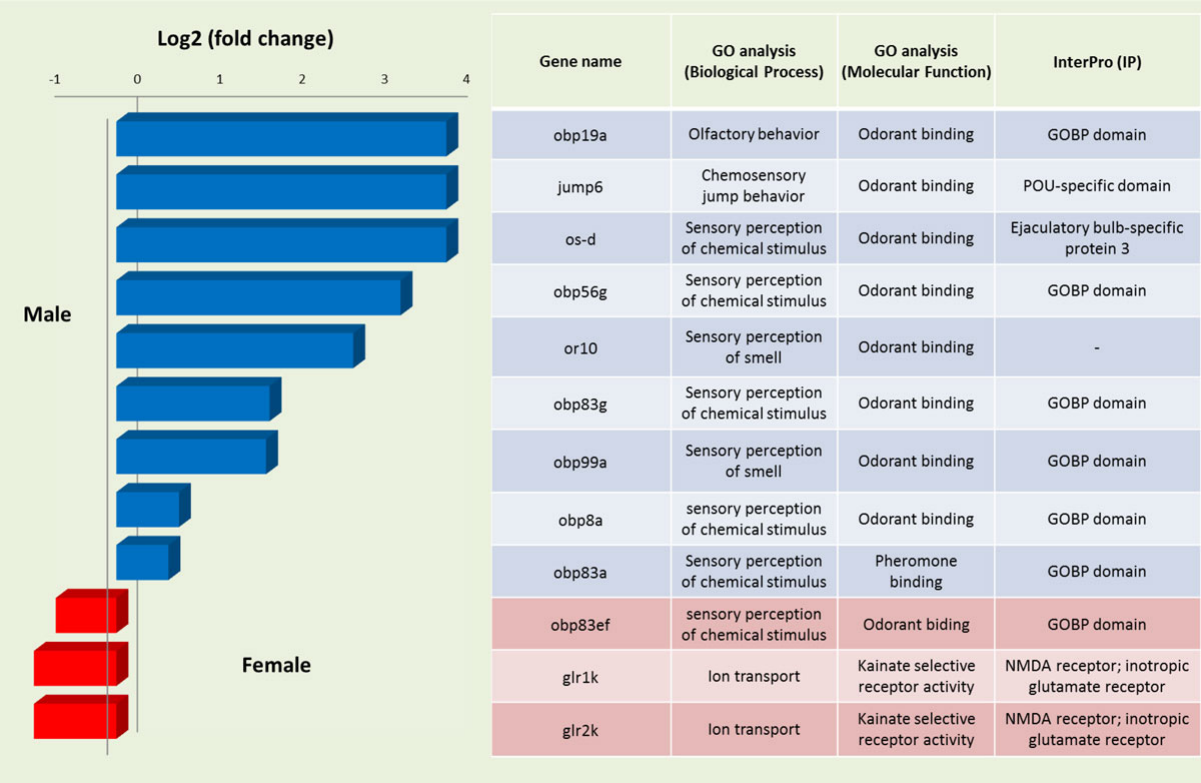
**Functional annotation of differentially expressed olfactory genes**. At the left part of the figure, the expression levels of the differentially expressed olfactory genes (Log2, fold change) are shown, as resulted from the RNA-seq analysis. The up-regulated genes in males are depicted in blue bars and the up-regulated genes in females in red bars. At the right part of the figure, the Gene Ontology (GO) classification of the same genes for the ontologies: Biological Process (BP), Molecular Function (MF) and Interpro (IP) protein domains is listed. Gene names are based on the nomenclature of the *Drosophila melanogaster *homologues [68].

With that in mind, we opted to explore the expression of various olfactory-related genes in the reproductive systems under investigation. Twelve olfactory-related genes were present in the annotated list that resulted from the transcriptome assembly of the FEMALE and MALE olive fly tissues (Figure 6), nine of which presented various levels of over-expression in MALE, whereas the remaining three in FEMALE. In order to get a deeper insight, the relative expression of five of these genes was further analyzed in female FAGs/spermathecae, male testes and male accessory glands (MAGs), before and after mating.

**Figure 7:**
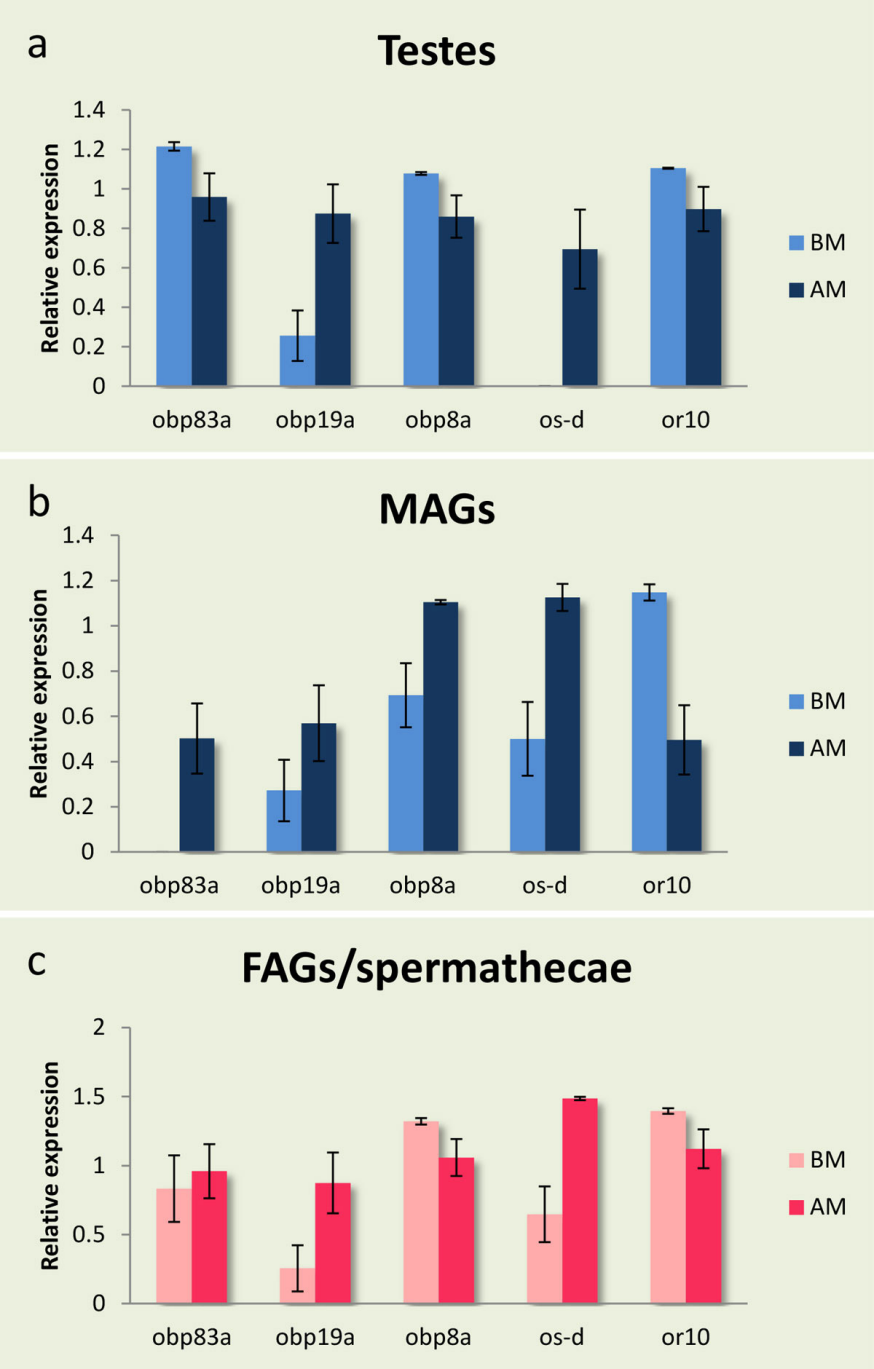
**Relative expression profiles of differentially expressed olfactory genes**. Expression profiles of five olfactory genes [odorant binding proteins *obp83a, obp19a, obp8a*, chemosensory protein, *os-d*, and odorant receptor 10, *or10*] as determined by qRT-PCR in three different tissues: Testes (a), MAGs (b) and FAGs/spermatheca (c) before (BM) and after (AM) mating. Standard error of the mean of five biological replicates is depicted in bars. No significant difference (for P < 0.05) was detected.

*obp83a, obp8a *and *obp19a *genes are over-expressed in MALE tissue (Figure 6). qRT-PCR revealed that these genes share the same expression pattern in MAGs. *obp83a and obp8a *are over-expressed before mating in testes while *obp83a *and *obp19a *are over-expressed after mating in FAGs/spermathecae (Figure 7). All three genes are characterized by a GOBP (general odorant binding protein) domain that is also found in their orthologues in *Drosophila melanogaster*. This structural domain is found in pheromone binding proteins, which exist in extracellular fluid surrounding odorant receptors [97]. The presence of these OBPs in the reproductive tissues implicates their interaction with other substrates except the olfactory system as transporters in the post-mating events in the male reproductive system. In fact, *D*. melanogaster's o*bp8a *shows the highest levels of expression in male accessory glands [98,99] and has been associated with non-olfactory functions such as RNA transcription [100].

*os-d *is over-expressed in MALE tissue (Figure 6) while qRT-PCR showed similar expression patterns in mature FAGs/spermathecae, MAGs and testes, but no expression in MAGs before mating (Figure 7). Os-D is a chemosensory protein (CSP) that encodes the antennal protein 10 in *D. melanogaster*. CSPs are secreted in the sensillum lymph of insect chemosensory sensilla and some OS-D-like proteins bind short to medium chain length fatty acid derivatives with low specificity [101,102]. Their specific function remains uncertain [103], suggesting a more general physiological function relating to the transport/solubility of hydrophobic ligands in various tissues.

*or10 *showed expression in male tissues (Figure 6) while qRT-PCR detected same transcriptional profiles in all three tissues before and after mating (Figure 7). *or10 *encodes an olfactory receptor protein and has a G-protein coupled receptor activity. The expression of ORs in testes has been reported for a number of species [90,104]. ORs' function in mammalian sperm is thought to regulate motility in response to exogenous signals derived from the existence of sperm-egg chemotaxis in invertebrates. The small peptides, speract and resact, are secreted by sea urchin eggs and attract spermatozoa in a species-specific manner by stimulating sperm motility and respiration [105,106]. The presence of a similar chemoreceptor may be essential in female spermatheca in order to establish a concentration gradient of a putative chemo-attractant. Since female accessory glands and spermatheca were dissected together, we are not able at this point to establish which exact tissue is the source of the observed expression of *or10*.

### 5. Early embryonic gene expression in the olive fly

As mentioned in the Background, promoters of early embryonic genes in combination with pro-apoptotic cell death genes are very important tools in inducing dominant early-embryonic lethality during insect transgenesis [107]. In that regard, the *serendipity-α *(*sry-α*) and *head involution defective *(*hid*) genes were selected for expression evaluation during embryonic development in the olive fly.

The embryonic developmental progress begins with the egg maturation and formation of the zygote, then enters the stage of blastoderm formation and gastrulation and ultimately ends with the organogenesis. Accordingly, three stages of embryogenesis have been also designated in *B. oleae*, whose average duration is 65-70h at 25 ± 1°C under standard laboratory conditions [108]. Microscopy morphological observations in living embryos report that cellularization of the blastoderm begins 6h after oviposition and lasts until 10h. During the third stage of organogenesis, the ventral furrow formation starts by 22h and the head and abdominal lobe masses become visible by 46h. Gut and mouth hook formation can be identified by 52h, whereas the development of other systems are distinct by 60h.

In *Drosophila melanogaster, sry-α *gene is specifically transcribed at the blastoderm stage in all somatic nuclei, from nuclear cycle 11 to the onset of gastrulation [109]. The gene product is required for the complete reorganization of the microfilaments at the onset of membrane invagination [110]. *sry-α *is fast evolving even within the Drosophilidae [111] and extensive divergence of many developmental genes within dipterans has also been reported [112-114]. This was most likely the reason for the unsuccessful efforts in *C. capitata *to obtain *sry-α *by degenerate PCR on the basis of sequence similarity with the homologous *D. melanogaster *[115]. Given the availability of both *D. melanogaster *and *C. capitata sry-α *sequences in the NCBI database, a homology search in the *B. oleae *transcriptome identified the relevant *B. oleae sry-α *gene homologue.

**Figure 8:**
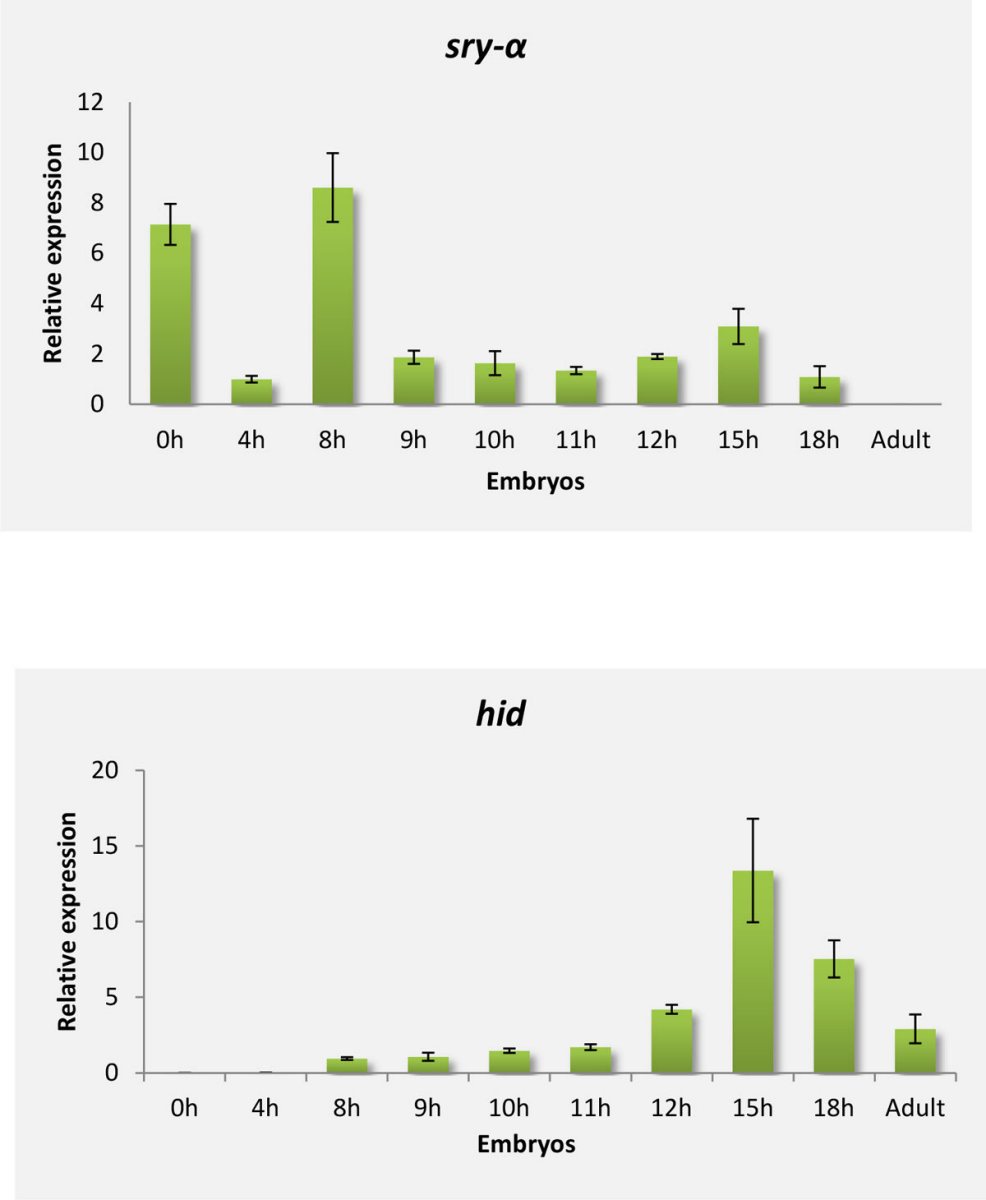
**Expression profile analysis during the early stages of embryogenesis**. Expression levels of Α) *Bosry-α *and B) *Bohid *in individual eggs collected at different time points during embryonic development, as determined by qRT-PCR. Standard error of the mean of two biological replicates per time point is depicted in bars.

Based on this sequence, *B. oleae*-specific primers were designed and the expression profiles of *sry-α *mRNA were studied by qRT-PCR analysis at different stages of *B. oleae *embryonic development. Eggs were collected throughout embryogenesis from the time of egg laying to larval hatching. The selected time points represented embryos at 0h, 4h, 8h, 9h, 10h, 11h, 12h, 15h and 18h after oviposition (Figure 8, panel A). This analysis revealed that *sry-α *mRNA is developmentally regulated during the second major event in the first stage of embryogenesis. It is initially present in large amounts just after oviposition (0h embryos), following a reduction in 4h embryos. The larger amounts of the transcripts among all time points examined were detected in 8h embryos. This suggests the presence of maternal mature transcripts which in turn are eliminated probably in the first event of maternal-to-zygotic transition (MZT). The subsequent wave of 'zygotic' activity requires zygotically synthesized transcripts [116]. In *D. melanogaster *as well as in *C. capitata, sry-α *is expressed only in the zygote [117]. However the retrieved *B. oleae *transcript shared greater amino acid similarity to the *D. melanogaster *CG8247 gene than to *sry-α*, as was also reported for the *Ccsry-α like *gene [118]. The orthologous CG8247 in *D. melanogaster *is characterized as a *sry-α*-like gene being also involved in cellular blastoderm formation. However, it is maternally inherited in contrast to *sry-α*, demonstrating a different mechanism of molecular control of transcription. In our case *Bosry-α like *gene seems to be maternally supplied in the embryos as mature transcripts. Previous studies have designated that the cellular blastoderm formation in *C. capitata *occurs within 9 h and 11 h after oviposition [115]. In accordance with *C. capitata*, a relative Tephritid species, we suggest that the cellurarization process in *B. oleae *during embryogenesis also occurs at 8h, since the *sry-α *transcripts were detected at higher levels during this time.

### 6. Apoptotic gene expression

At the same time, *head involution defective *(*hid*), known to have a central role in apoptosis pathway, was also selected for further study. Apoptosis is a genetically controlled mechanism of cytological events that results in programmed cell death. During development, programmed cell death plays a key role by eliminating unwanted cells from a variety of tissues, such as, for example, larval tissues during insect metamorphosis (Reviewed in [119]). A series of caspases, a family of cysteine proteases, play a central role during apoptosis. Once activated, caspases can cleave more than 100 different cell target proteins, bringing about ultimately the cell death [120]. Regulators of caspase activation may either promote apoptosis (pro-apoptotic) or inhibit apoptosis (anti-apoptotic). Drosophila Hid belongs to a family of pro-apoptotic proteins which act as antagonists of IAPs (Inhibitor of Apoptosis Proteins), thus resulting in caspase activation and apoptosis [119,121,122]. Such pro-apoptotic genes have been used in transgenic control systems for pest insects. In tetracycline-suppressible systems for female-specific lethality and conditional embryonic expression of a Drosophila *hid*-containing transgene, for example, 100% lethality was observed in Drosophila [123], as well as in the Tephritid flies *Ceratitis capitata *[117] and *Anastrepha suspensa *[124].

The developmental regulation of *Bohid *was explored by determining the transcript levels during embryogenesis. A qRT-PCR approach with species-specific primers was used to evaluate the expression pattern of *hid *in embryos at 0 h, 4 h, 8 h, 9 h, 10 h, 11 h, 12 h, 15 h, 18 h after oviposition. Based on *D. melanogaster hid *expression pattern, no expression was expected in embryos prior to formation of the syncytial blastoderm [125]. Indeed, until 8h no transcripts were detected. *hid *expression was first detected at 12h and peaked during 15h (Figure 8, panel B).

It is noteworthy that most developmental programmed cell death occurs during the gastrulation process of *D. melanogaster *embryonic development [126], suggesting that the onset of this period in *B. oleae *could be defined approximately at 12h, occurring mainly within 15-18h.

However, further examination of the pro-apoptotic function of *hid *gene is required in order to explore its ability of inducing apoptosis in *B. oleae *cells. Specific lethal embryonic phenotypes need to be obtained to characterize its role in the cell-death pathway. Ongoing analysis for the isolation of the complete gene will provide the essential tools for the generation of an endogenous effective lethal effector system.

## Conclusions

In serious agricultural pests (like the olive fly) which are not model experimental organisms (unlike the medfly), the major focus of most scientific research is, in the end, directed towards control of the pest. Old and new environmental concerns and sensibilities, that regard mostly insecticide use, drive science to the quest of alternative, environmentally friendlier methods of pest control. Time and again it has been shown that such methods go through thorough understanding of the biology and ecology of the target organism. Since the initial unsuccessful SIT efforts, molecular and genetic studies in the olive fly have focused on genetic analyses of natural populations, cytogenetics, isolation and characterization of genes that control important biological processes, as well as the identification and mapping of several microsatellite loci. Just a few years ago, *B. oleae *was successfully transformed, an achievement that gave new perspective towards the efficient use of the SIT. Lately, this is being coupled with genomics studies and transcriptomics analyses of various important systems, as well as efforts in advancing olive fly mass-rearing, that are setting the ground for the application of modern control approaches through the genetic manipulation of the insect.

## Methods

### Ethics statement

The study was carried out on laboratory reared olive flies. No specific permissions are required for these experiments, since these studies did not involve endangered or protected species.

### Fly culture and stocks

#### Laboratory strain

The laboratory strain of the olive fly (LAB) is part from the original stock from the Department of Biology, 'Demokritos' Nuclear Research Centre, Athens, Greece, and has been reared in our laboratory for over 15 years. The flies are reared at 25°C with a 12h light/12h dark photoperiod in 30x30x30cm^3 ^cages, as described by [127-129].

#### Egg collection

For embryo analysis, eggs were collected from 10-day old mated females maintained in our laboratory, which were fed with artificial adult diet to ensure high oviposition rates and embryo viability. Adults were exposed to paraffin oviposition domes for 10 minutes and the eggs were obtained with a 0.3% propionic acid solution, assigning this as the start time point. Eggs were maintained in an incubator according to the standard rearing conditions.

### RNA isolation for library preparation and functional analysis

Total RNA was isolated from female accessory glands (FAGs) and spermathecae of ~300 female flies and from testes of ~150 male flies. Four-day old sexually immature unmated insects were used. For RNA isolation, the TRIzol^® ^Reagent (Ambion-Invitrogen) was used, following the instructions of the manufacturer with minor modifications. RNA extraction was followed by an additional DNA removal using the TURBO DNA-free Kit (Ambion-Invitrogen), according to manufacturer's instructions. The integrity of RNA was assessed by 1% agarose gel electrophoresis and the purity of all RNA samples was evaluated at Fleming Institute (Greece) with the use of (Agilent 2100 Bioanalyzer) and NanoDrop (2000).

### Whole transcriptome library preparation for next-generation sequencing with the SOLiD 4 Sequencing System

RNA transcripts from olive fly FAGs/spermathecae (FEMALE) and testes (MALE) were used to construct two cDNA libraries for sequencing analysis on the SOLiD 4 Sequencing System. More specifically, polyadenylated RNA (polyA-RNA) was isolated from 5 μg of total RNA using the Dynabeads Oligo(dT) kit (Ambion, Life Technologies Corporation). The isolated polyA-RNA was randomly fragmented by chemical hydrolysis at 94°C for 5 minutes and was then treated with antarctic phosphatase to remove phosphate groups from the fragments' ends, followed by treatment with T4 polynucleotide kinase to add a Pi at the 5' end of each fragment. The resulting RNA fragments were hybridized and ligated to the P1 and P2 adaptor sequences specifically designed for sequencing with the SOLiD system (SOLiD Total RNA-Seq Kit, Life Technologies Corporation). The RNA produced was reverse transcribed to cDNA which was then amplified in a 15-cycle PCR. At this step, the use of different barcoded 3' PCR primers from the selection included in the SOLiD barcoding kit allowed the preparation of cDNA libraries for multiplex sequencing. From the cDNA produced, only fragments of average size 200-300 bp were selected with two rounds of magnetic bead purification (Agencourt AMPure XP Reagent, Beckman Coulter).

The quality and size of the purified cDNA library was assessed on the Agilent Bioanalyzer 2100 (Agilent Technologies Inc.) and with quantitative PCR using the Library Quant Kit ABI Solid (KAPA Biosystems). A multiplex library mix (500pM) was used to prepare a full-slide for analysis on the SOliD 4 Sequencing System (Applied Biosystems) with 35+50 bp PE-chemistry.

### RNA isolation and expression analysis of selected genes

*RNA extraction for expression analysis of sexually differentially expressed genes*. For the validation of the differential expression of sexually differentially expressed genes, RNA was extracted from two pools of 40 pairs of spermathecae/FAGs and 40 pairs of testes (two biological pool replicates), dissected from an equivalent number of female and male adult laboratory flies, respectively.

*RNA extraction for expression analysis of olfactory and early embryonic developmental genes*. For the validation of the olfactory genes expression, RNA was extracted from five female and five male individual insects (five biological replicates, respectively) before and after mating of the aforementioned laboratory strain. Two groups of insects were considered. Firstly, unmated insects, i.e., sexually mature 7-day old unmated insects (before mating, BM). Secondly, mated insects, i.e., sexually mature 7-day old insects that were allowed to mate on the seventh day and were dissected 12 hours after mating (after mating, AM). For the validation of the sexually differentially expressed genes, the RNA isolated for the construction of the two libraries was used. RNA was extracted using TriZol reagent according to manufacturer's protocol.

For the validation of the early embryonic genes, eggs were removed from the incubator at different time intervals throughout embryonic development and total RNA was extracted from each egg using TriZol reagent according to the manufacturer's protocol. Two individual eggs (two biological replicates) from the various time points during the embryonic developmental stages were used for the extractions.

Following extraction, the RNA was treated with 1.0 unit of DNase I (Invitrogen) according to manufacturer's instructions. In all of the above cases, the total amount of DNA-free RNA obtained from each tissue (between 400 to 700 ng) was converted into cDNA using 300ng Random hexamer primers (equimolar mix of N_5_A, N_5_G, N_5_C and N_5_T), 200 units MMLV Reverse Transcriptase (Geneon), 5X reaction buffer, 40mM dNTP mix and 40 units RNase Inhibitor (GeneOn) according to the manufacturer's instructions. Reverse transcription was conducted at 42°C for 50 min and 70°C for 15 min. The resulting cDNA was used in the subsequent qPCR reactions.

Specific primers for the amplification of selected differentially expressed genes revealed by the transcriptome analysis were designed by Primer-BLAST (http://www.ncbi.nlm.nih.gov/tools/primer-blast) (Table S2). To identify sequences with homology to the genes *sry-α *and *hid*, the orthologous genes of *C. capitata *and *An. suspensa *were used as queries to search for *B. oleae *transcripts using tBLASTX in the TSA Database. Species-specific Blast hits for each of the query sequences were retrieved (Genbank: GAKB01005111.1, GAKB01003654.1) and used to design primers (Table S2) for the subsequent amplification of gene-specific sequences by quantitative real-time PCR (qRT-PCR).

Relative quantitation was used to analyze changes in expression levels of the selected genes using a Real-time PCR approach. Expression values were calculated relatively to the housekeeping *rpl19 *gene. *Rpl19 *and *14-3-3z *genes were used as reference in MAGs and testes while *actin3 *and *a-tubulin *in FAGs/spermathecae. The qRT-PCR conditions were: polymerase activation and DNA denaturation step at 95 °C for 4 min, followed by 40 cycles of denaturation at 95 °C for 30 s, annealing/extension and plate read at 56 °C for 30 s and finally, a step of melting curve analysis at a gradual increase of temperature over the range 55 °C → 95 °C. In this step, the detection of one gene specific peak and the absence of primer dimer peaks was assured. Each reaction was performed in a total volume of 15 μl, containing 5 μl from a dilution 1:10 of the cDNA template, 1X iTaq Universal SYBR Green Supermix (Biorad, Gaithesburg, MD) and 400nM of each primer. The reactions were carried out on Bio-Rad Real-Time thermal cycler CFX96 (Bio-Rad, Hercules, CA, USA) and data analysed using the CFX Manager™ software. All qRT-PCRs were performed in triplicate (i.e., three technical replicates).

### Bioinformatics analysis

All paired and unpaired reads of the libraries were assembled to construct the reference transcriptome using the SOAPdenovo assembler [54] with a word size of 25 nt. Annotation of the assembled sequences was obtained by comparing to the NCBI non-redundant (Nr) protein database (May 7^th^, 2014 version) using blastx [130] and collecting the annotations with the BLAST2GO tool [55]. TopHat [131] was used to generate a spliced alignment to the reference transcriptome. Transcripts were assembled using Cufflinks and differentially expressed genes were identified using Cuffdiff [56]. GO-term enrichment between male and female transcriptomes was analyzed using the using the GOSSIP [132] application embedded in BLAST2GO.

## Availability of supporting data

The data sets supporting the results of this article are included within the article and its additional files. Additional File 1, Additional File 2, Additional File 3 and Additional File 4

## Competing interests

The authors declare that they have no competing interests.

## Authors' contributions

ES was involved in the transcriptome library construction and performed the analysis of the sex-determination genes; MR performed the bioinformatics analysis of the transcriptome; VH constructed the transcriptome libraries and analysed the sequencing data; KTT and AMM analyzed the embryonic and apoptotic genes; MEG, ST and KA analysed the olfactory genes; JR directed the bioinformatics analysis; KDM designed and coordinated the study. All authors participated in drafting the manuscript and read and approved the final document.

## Supplementary Material

Additional File 1 Click here for file

Additional File 1 Click here for file

Additional File 1 Click here for file

Additional File 1 Click here for file

## References

[B1] Theophrastus Enquiry into plants (History of plants HP), I & II (HORT, A. F., translator) 1916 London, Cambridge & Massachusetts [in ancient Greek with English translation]

[B2] Daane KM Johnson MW Olive fruit fly: managing an ancient pest in modern times Annu Rev Entomol 2010 55 151 69 10.1146/annurev.ento.54.110807.090553 19961328

[B3] Pimentel D Ecological Effects of Pesticides on Non-target Species 1971 Washington, D.C.: Executive Office of the President, Office of Science and Technology 220

[B4] Baumhover A Graham A Bitter B Hopkins D New W Dudleyandr F Bushland C Screwworm control through release of sterilized flies J Econ Entomol 1955 462 466

[B5] Knipling E Possibilities of insect control or eradication through the use of sexually sterile males J Econ Entomol 1955 459 462

[B6] Greek Ministry of Agriculture Description of research organization for the control of the olive fruit fly 1961 33 (in Greek)

[B7] Economopoulos A Avtzis N Zervas G Tsitsipis J Haniotakis G Tsiropoulos G Manoukas A Control of the olive fly, Dacus oleae (Gmelin), by the combined effects of insecticides and release of gamma sterilized insects J Appl Entomol 1977 201 215

[B8] Economopoulos AP Haniotakis GE Mathioudis J Missis N Kinigakis P Long-distance flight of wild and artificially-reared Dacus oleae (Gmelin) (Diptera, Tephritidae) Z Angew Entomol 1978 101 108

[B9] Economopoulos A Zervas G The quality problem in olive flies produced for SIT experiments IAEA STI/PUB 1982

[B10] Economopoulos A The olive fruit fly, Bactrocera (Dacus) oleae (Gmelin) (Diptera: Tephritidae): its importance and control; previous SIT research and pilot testing Int At Energy Agency, Vienna, Austria 2002

[B11] Zervas GA Economopoulos AP Mating frequency in caged populations of wild and artificially reared (normal or γ-sterilized) olive fruit flies Environ Entomol 1982 17 20

[B12] Loukas M Economopoulos AP Zouros E Vergini Y Genetic changes in artificially reared colonies of the olive fruit fly Ann Ent Soc Amer 1985 159 165

[B13] Economopoulos A Loukas M ADH allele frequency changes in olive fruit flies shift from olives to artificial larval food and vice versa, effect of temperature Entomol Exp Appl 1986 215 221

[B14] Economopoulos A Sexual competitiveness of gamma-ray sterilized males of Dacus oleae. Mating frequency of artificially reared and wild females Env Entomol 1972 490 497

[B15] Capuzzo C Firrao G Mazzon L Squartini A Girolami V "Candidatus Erwinia dacicola", a coevolved symbiotic bacterium of the olive fly Bactrocera oleae (Gmelin) Int J Syst Evol Microbiol 2005 55 Pt 4 1641 7 16014495 10.1099/ijs.0.63653-0

[B16] Sacchetti P Granchietti A Landini S Viti C Giovannetti L Belcari A Relationships between the olive fly and bacteria J Appl Entomol 2008 132 682 689 10.1111/j.1439-0418.2008.01334.x

[B17] Estes AM Hearn DJ Bronstein JL Pierson EA The olive fly endosymbiont, "Candidatus Erwinia dacicola," switches from an intracellular existence to an extracellular existence during host insect development Appl Environ Microbiol 2009 75 7097 106 10.1128/AEM.00778-09 19767463 PMC2786516

[B18] Ben-Yosef M Aharon Y Jurkevitch E Yuval B Give us the tools and we will do the job: symbiotic bacteria affect olive fly fitness in a diet-dependent fashion Proc Biol Sci 2010 277 1545 52 10.1098/rspb.2009.2102 20071385 PMC2871834

[B19] Kounatidis I Crotti E Sapountzis P Sacchi L Rizzi A Chouaia B Bandi C Alma A Daffonchio D Mavragani-Tsipidou P Bourtzis K Acetobacter tropicalis is a major symbiont of the olive fruit fly (Bactrocera oleae) Appl Environ Microbiol 2009 75 3281 8 10.1128/AEM.02933-08 19304818 PMC2681620

[B20] Augustinos AA Stratikopoulos EE Zacharopoulou A Mathiopoulos KD Polymorphic microsatellite markers in the olive fly, Bactrocera oleae Mol Ecol Notes 2002 2 278 280 10.1046/j.1471-8286.2002.00222.x

[B21] Nardi F Carapelli A Dallai R Frati F The mitochondrial genome of the olive fly Bactrocera oleae: two haplotypes from distant geographical locations Insect Mol Biol 2003 12 605 611 10.1046/j.1365-2583.2003.00445.x 14986921

[B22] Augustinos AA Mamuris Z Stratikopoulos EE D'Amelio S Zacharopoulou A Mathiopoulos KD Microsatellite analysis of olive fly populations in the Mediterranean indicates a westward expansion of the species Genetica 2005 125 231 41 10.1007/s10709-005-8692-y 16247695

[B23] Nardi F Carapelli A Dallai R Roderick GK Frati F Population structure and colonization history of the olive fly, Bactrocera oleae (Diptera, Tephritidae) Mol Ecol 2005 14 2729 38 10.1111/j.1365-294X.2005.02610.x 16029474

[B24] Nardi F Carapelli A Boore JL Roderick GK Dallai R Frati F Domestication of olive fly through a multi-regional host shift to cultivated olives: comparative dating using complete mitochondrial genomes Mol Phylogenet Evol 2010 57 678 86 10.1016/j.ympev.2010.08.008 20723608

[B25] Zygouridis NE Augustinos AA Zalom FG Mathiopoulos KD Analysis of olive fly invasion in California based on microsatellite markers Heredity (Edinb) 2009 102 402 12 10.1038/hdy.2008.125 19107137

[B26] Dogaç E Kandemir İ Taskin V The genetic polymorphisms and colonization process of olive fly populations in Turkey PLoS One 2013 8 e56067 10.1371/journal.pone.0056067 23457499 PMC3573072

[B27] Mavragani-Tsipidou P Genetic and cytogenetic analysis of the olive fruit fly Bactrocera oleae (Diptera: Tephritidae) Genetica 2002 116 45 57 10.1023/A:1020907624816 12484525

[B28] Mavragani-Tsipidou P Karamanlidou G Zacharopoulou A Koliais S Kastritisis C Mitotic and polytene chromosome analysis in Dacus oleae (Diptera: Tephritidae) Genome 1992 35 373 8 10.1139/g92-056 1624130

[B29] Zambetaki A Kleanthous K Mavragani-Tsipidou P Cytogenetic analysis of Malpighian tubule and salivary gland polytene chromosomes of Bactrocera oleae (Dacus oleae) (Diptera: Tephritidae) Genome 1995 38 1070 81 10.1139/g95-143 18470232

[B30] Drosopoulou E Chrysopoulou A Nikita V Mavragani-Tsipidou P The heat shock 70 genes of the olive pest Bactrocera oleae: genomic organization and molecular characterization of a transcription unit and its proximal promoter region Genome 2009 52 210 4 10.1139/G08-110 19234568

[B31] Drosopoulou E Nakou I Síchová J Kubíčková S Marec F Mavragani-Tsipidou P Sex chromosomes and associated rDNA form a heterochromatic network in the polytene nuclei of Bactrocera oleae (Diptera: Tephritidae) Genetica 2012 140 169 80 10.1007/s10709-012-9668-3 22825842

[B32] Vontas JG Hejazi MJ Hawkes NJ Cosmidis N Loukas M Hemingway J Janes RW Resistance-associated point mutations of organophosphate insensitive acetylcholinesterase, in the olive fruit fly Bactrocera oleae Insect Mol Biol 2002 11 329 336 April 10.1046/j.1365-2583.2002.00343.x 12144698

[B33] Vontas J Blass C Koutsos AC David JP Kafatos FC Louis C Hemingway J Christophides GK Ranson H Gene expression in insecticide resistant and susceptible Anopheles gambiae strains constitutively or after insecticide exposure Insect Mol Biol 2005 14 509 21 10.1111/j.1365-2583.2005.00582.x 16164607

[B34] Kakani EG Mathiopoulos KD Organophosphosphate resistance-related mutations in the acetylcholinesterase gene of Tephritidae J Appl Entomol 2008 132 762 771 10.1111/j.1439-0418.2008.01373.x

[B35] Kakani EG Bon S Massoulié J Mathiopoulos KD Altered GPI modification of insect AChE improves tolerance to organophosphate insecticides Insect Biochem Mol Biol 2011 41 150 8 10.1016/j.ibmb.2010.11.005 21112395

[B36] Khila A El Haidani A Vincent A Payre F Souda SI The dual function of ovo/shavenbaby in germline and epidermis differentiation is conserved between Drosophila melanogaster and the olive fruit fly Bactrocera oleae Insect Biochem Mol Biol 2003 33 691 9 10.1016/S0965-1748(03)00063-8 12826096

[B37] Benos P Tavernarakis N Brogna S Thireos G Savakis C Acquisition of a potential marker for insect transformation: isolation of a novel alcohol dehydrogenase gene from Bactrocera oleae by functional complementation in yeast Mol Gen Genet 2000 263 90 5 10.1007/PL00008679 10732677

[B38] Lagos D Ruiz MF Sánchez L Komitopoulou K Isolation and characterization of the Bactrocera oleae genes orthologous to the sex determining Sex-lethal and doublesex genes of Drosophila melanogaster Gene 2005 348 111 21 15777677 10.1016/j.gene.2004.12.053

[B39] Lagos D Koukidou M Savakis C Komitopoulou K The transformer gene in Bactrocera oleae: the genetic switch that determines its sex fate Insect Mol Biol 2007 16 221 30 10.1111/j.1365-2583.2006.00717.x 17298554

[B40] Tsoumani KT Mathiopoulos KD Genome size estimation with quantitative real-time PCR in two Tephritidae species: Ceratitis capitata and Bactrocera oleae J Appl Entomol 2012 136 626 631 10.1111/j.1439-0418.2011.01684.x

[B41] Tsoumani KT Drosopoulou E Mavragani-Tsipidou P Mathiopoulos KD Molecular characterization and chromosomal distribution of a species-specific transcribed centromeric satellite repeat from the olive fruit fly, Bactrocera oleae PLoS One 2013 8 e79393 10.1371/journal.pone.0079393 24244494 PMC3828357

[B42] Tsoumani KT Augustinos AA Kakani EG Drosopoulou E Mavragani-Tsipidou P Mathiopoulos KD Isolation, annotation and applications of expressed sequence tags from the olive fly, Bactrocera oleae Mol Genet Genomics 2011 285 33 45 10.1007/s00438-010-0583-y 20978910

[B43] Pavlidi N Dermauw W Rombauts S Chrisargiris A Van Leeuwen T Vontas J Analysis of the Olive Fruit Fly Bactrocera oleae Transcriptome and Phylogenetic Classification of the Major Detoxification Gene Families PLoS One 2013 8 e66533 10.1371/journal.pone.0066533 23824998 PMC3688913

[B44] Koukidou M Klinakis A Reboulakis C Zagoraiou L Tavernarakis N Livadaras I Economopoulos A Savakis C Germ line transformation of the olive fly Bactrocera oleae using a versatile transgenesis marker Insect Mol Biol 2006 15 95 103 10.1111/j.1365-2583.2006.00613.x 16469073

[B45] Ant T Koukidou M Rempoulakis P Gong HF Economopoulos A Vontas J Alphey L Control of the olive fruit fly using genetics-enhanced sterile insect technique BMC Biol 2012 10 51 10.1186/1741-7007-10-51 22713628 PMC3398856

[B46] Apostolaki A Livadaras I Saridaki A Chrysargyris A Savakis C Bourtzis K Transinfection of the olive fruit fly Bactrocera oleae with Wolbachia: towards a symbiont-based population control strategy J Appl Entomol 2011 135 546 553 10.1111/j.1439-0418.2011.01614.x

[B47] Estes AM Hearn DJ Burrack HJ Rempoulakis P Pierson EA Prevalence of Candidatus Erwinia dacicola in wild and laboratory olive fruit fly populations and across developmental stages Environ Entomol 2012 41 265 74 10.1603/EN11245 22506998

[B48] Alphey L Andreasen M Dominant lethality and insect population control Mol Biochem Parasitol 2002 121 173 8 10.1016/S0166-6851(02)00040-3 12034450

[B49] Alphey L Beard C Ben Billingsley P Coetzee M Crisanti A Curtis C Eggleston P Godfray C Hemingway J Jacobs-Lorena M James AA Kafatos FC Mukwaya LG Paton M Powell JR Schneider W Scott TW Sina B Sinden R Sinkins S Spielman A Touré Y Collins FH Malaria control with genetically manipulated insect vectors Science 2002 298 119 21 10.1126/science.1078278 12364786

[B50] Heinrich JC Scott MJ A repressible female-specific lethal genetic system for making transgenic insect strains suitable for a sterile-release program Proc Natl Acad Sci USA 2000 97 8229 32 10.1073/pnas.140142697 10890889 PMC26929

[B51] Thomas DD Donnelly CA Wood RJ Alphey LS Insect population control using a dominant, repressible, lethal genetic system Science 2000 287 2474 6 10.1126/science.287.5462.2474 10741964

[B52] Gong P Epton MJ Fu G Scaife S Hiscox A Condon KC Condon GC Morrison NI Kelly DW Dafa'alla T Coleman PG Alphey L A dominant lethal genetic system for autocidal control of the Mediterranean fruitfly Nat Biotechnol 2005 23 453 6 10.1038/nbt1071 15750586

[B53] Sagri E Reczko M Gregoriou M-E Tsoumani KT Zygouridis NE Salpea KD Zalom FG Ragoussis J Mathiopoulos KD Olive fly transcriptomics analysis implicates energy metabolism genes in spinosad resistance BMC Genomics 2014 15 714 10.1186/1471-2164-15-714 25156405 PMC4168201

[B54] Li R Zhu H Ruan J Qian W Fang X Shi Z Li Y Li S Shan G Kristiansen K Li S Yang H Wang J Wang J De novo assembly of human genomes with massively parallel short read sequencing Genome Res 2010 20 265 72 10.1101/gr.097261.109 20019144 PMC2813482

[B55] Götz S García-Gómez JM Terol J Williams TD Nagaraj SH Nueda MJ Robles M Talón M Dopazo J Conesa A High-throughput functional annotation and data mining with the Blast2GO suite Nucleic Acids Res 2008 36 3420 35 10.1093/nar/gkn176 18445632 PMC2425479

[B56] Trapnell C Williams BA Pertea G Mortazavi A Kwan G van Baren MJ Salzberg SL Wold BJ Pachter L Transcript assembly and quantification by RNA-Seq reveals unannotated transcripts and isoform switching during cell differentiation Nat Biotechnol 2010 28 511 5 10.1038/nbt.1621 20436464 PMC3146043

[B57] Domanitskaya E V Liu H Chen S Kubli E The hydroxyproline motif of male sex peptide elicits the innate immune response in Drosophila females FEBS J 2007 274 5659 68 10.1111/j.1742-4658.2007.06088.x 17922838

[B58] McGraw LA Clark AG Wolfner MF Post-mating gene expression profiles of female Drosophila melanogaster in response to time and to four male accessory gland proteins Genetics 2008 179 1395 408 10.1534/genetics.108.086934 18562649 PMC2475742

[B59] Hardy RW Tokuyasu KT Lindsley DL Analysis of spermatogenesis in Drosophila melanogaster bearing deletions for Y-chromosome fertility genes Chromosoma 1981 83 593 617 10.1007/BF00328522 6794995

[B60] Gibbons IR Dynein family of motor proteins: present status and future questions Cell Motil Cytoskeleton 1995 32 136 44 10.1002/cm.970320214 8681396

[B61] Goldstein LS Hardy RW Lindsley DL Structural genes on the Y chromosome of Drosophila melanogaster Proc Natl Acad Sci USA 1982 79 7405 9 10.1073/pnas.79.23.7405 6818544 PMC347348

[B62] Gepner J Hays TS A fertility region on the Y chromosome of Drosophila melanogaster encodes a dynein microtubule motor Proc Natl Acad Sci USA 1993 90 11132 6 10.1073/pnas.90.23.11132 8248219 PMC47936

[B63] Koerich LB Wang X Clark AG Carvalho AB Low conservation of gene content in the Drosophila Y chromosome Nature 2008 456 949 51 10.1038/nature07463 19011613 PMC2713029

[B64] Carvalho AB Lazzaro BP Clark AG Y chromosomal fertility factors kl-2 and kl-3 of Drosophila melanogaster encode dynein heavy chain polypeptides Proc Natl Acad Sci USA 2000 97 13239 44 10.1073/pnas.230438397 11069293 PMC27209

[B65] Kimble J Edgar L Hirsh D Specification of male development in Caenorhabditis elegans: the fem genes Dev Biol 1984 105 234 9 10.1016/0012-1606(84)90279-3 6468762

[B66] Doniach T Hodgkin J A sex-determining gene, fem-1, required for both male and hermaphrodite development in Caenorhabditis elegans Dev Biol 1984 106 223 35 10.1016/0012-1606(84)90077-0 6541600

[B67] Spence AM Coulson A Hodgkin J The product of fem-1, a nematode sex-determining gene, contains a motif found in cell cycle control proteins and receptors for cell-cell interactions Cell 1990 60 981 90 10.1016/0092-8674(90)90346-G 2317869

[B68] flybase http://www.flybase.org

[B69] Yeh SD Chen YJ Chang AC Ray R She BR Lee WS Chiang HS Cohen SN Lin-Chao S Isolation and properties of Gas8, a growth arrest-specific gene regulated during male gametogenesis to produce a protein associated with the sperm motility apparatus J Biol Chem 2002 277 6311 7 10.1074/jbc.M106941200 11751847

[B70] Yang Y Cochran DA Gargano MD King I Samhat NK Burger BP Sabourin KR Hou Y Awata J Parry DAD Marshall WF Witman GB Lu X Regulation of flagellar motility by the conserved flagellar protein CG34110/Ccdc135/FAP50 Mol Biol Cell 2011 22 976 87 10.1091/mbc.E10-04-0331 21289096 PMC3069022

[B71] Garrett-Engele CM Siegal ML Manoli DS Williams BC Li H Baker BS intersex, a gene required for female sexual development in Drosophila, is expressed in both sexes and functions together with doublesex to regulate terminal differentiation Development 2002 129 4661 75 12361959 10.1242/dev.129.20.4661

[B72] Gubbay J Collignon J Koopman P Capel B Economou A Münsterberg A Vivian N Goodfellow P Lovell-Badge R A gene mapping to the sex-determining region of the mouse Y chromosome is a member of a novel family of embryonically expressed genes Nature 1990 346 245 50 10.1038/346245a0 2374589

[B73] Wilson MJ Dearden PK Evolution of the insect Sox genes BMC Evol Biol 2008 8 120 10.1186/1471-2148-8-120 18439299 PMC2386450

[B74] Crémazy F Berta P Girard F Sox neuro, a new Drosophila Sox gene expressed in the developing central nervous system Mech Dev 2000 93 215 9 10.1016/S0925-4773(00)00268-9 10781960

[B75] Prokopenko SN Brumby A O'Keefe L Prior L He Y Saint R Bellen HJ A putative exchange factor for Rho1 GTPase is required for initiation of cytokinesis in Drosophila Genes Dev 1999 13 2301 14 10.1101/gad.13.17.2301 10485851 PMC316993

[B76] O'Keefe L Somers WG Harley A Saint R The pebble GTP exchange factor and the control of cytokinesis Cell Struct Funct 2001 26 619 26 10.1247/csf.26.619 11942617

[B77] Khurana B Kristie TM A protein sequestering system reveals control of cellular programs by the transcriptional coactivator HCF-1 J Biol Chem 2004 279 33673 83 10.1074/jbc.M401255200 15190068

[B78] Talbo G Højrup P Rahbek-Nielsen H Andersen SO Roepstorff P Determination of the covalent structure of an N- and C-terminally blocked glycoprotein from endocuticle of Locusta migratoria. Combined use of plasma desorption mass spectrometry and Edman degradation to study post-translationally modified proteins Eur J Biochem 1991 195 495 504 10.1111/j.1432-1033.1991.tb15730.x 1997327

[B79] Pondeville E Maria A Jacques J-C Bourgouin C Dauphin-Villemant C Anopheles gambiae males produce and transfer the vitellogenic steroid hormone 20-hydroxyecdysone to females during mating Proc Natl Acad Sci USA 2008 105 19631 6 10.1073/pnas.0809264105 19060216 PMC2604965

[B80] Doctor J Fristrom D Fristrom JW The pupal cuticle of Drosophila: biphasic synthesis of pupal cuticle proteins in vivo and in vitro in response to 20-hydroxyecdysone J Cell Biol 1985 101 189 200 10.1083/jcb.101.1.189 3891759 PMC2113631

[B81] Leal WS Odorant reception in insects: roles of receptors, binding proteins, and degrading enzymes Annu Rev Entomol 2013 58 373 91 10.1146/annurev-ento-120811-153635 23020622

[B82] Pelosi P Maida R Odorant-binding proteins in insects Comp Biochem Physiol B Biochem Mol Biol 1995 111 503 14 10.1016/0305-0491(95)00019-5 7613772

[B83] Vosshall LB Hansson BS A unified nomenclature system for the insect olfactory coreceptor Chem Senses 2011 36 497 8 10.1093/chemse/bjr022 21441366

[B84] Vanderhaeghen P Schurmans S Vassart G Parmentier M Olfactory receptors are displayed on dog mature sperm cells J Cell Biol 1993 123 6 Pt 1 1441 52 8253843 10.1083/jcb.123.6.1441 PMC2290870

[B85] Vanderhaeghen P Schurmans S Vassart G Parmentier M Specific repertoire of olfactory receptor genes in the male germ cells of several mammalian species Genomics 1997 39 239 46 10.1006/geno.1996.4490 9119360

[B86] Kang N Koo J Olfactory receptors in non-chemosensory tissues BMB Rep 2012 45 612 22 10.5483/BMBRep.2012.45.11.232 23186999 PMC4133803

[B87] Spehr M Gisselmann G Poplawski A Riffell JA Wetzel CH Zimmer RK Hatt H Identification of a testicular odorant receptor mediating human sperm chemotaxis Science 2003 299 2054 8 10.1126/science.1080376 12663925

[B88] Spehr M Schwane K Heilmann S Gisselmann G Hummel T Hatt H Dual capacity of a human olfactory receptor Curr Biol 2004 14 R832 3 10.1016/j.cub.2004.09.034 15458659

[B89] Spehr M Schwane K Riffell JA Zimmer RK Hatt H Odorant receptors and olfactory-like signaling mechanisms in mammalian sperm Mol Cell Endocrinol 2006 250 128 36 10.1016/j.mce.2005.12.035 16413109

[B90] Fukuda N Yomogida K Okabe M Touhara K Functional characterization of a mouse testicular olfactory receptor and its role in chemosensing and in regulation of sperm motility J Cell Sci 2004 117 Pt 24 5835 45 15522887 10.1242/jcs.01507

[B91] Veitinger T Riffell JR Veitinger S Nascimento JM Triller A Chandsawangbhuwana C Schwane K Geerts A Wunder F Berns MW Neuhaus EM Zimmer RK Spehr M Hatt H Chemosensory Ca2+ dynamics correlate with diverse behavioral phenotypes in human sperm J Biol Chem 2011 286 17311 25 10.1074/jbc.M110.211524 21454470 PMC3089573

[B92] Pitts RJ Liu C Zhou X Malpartida JC Zwiebel LJ Odorant receptor-mediated sperm activation in disease vector mosquitoes Proc Natl Acad Sci USA 2014 111 2566 71 10.1073/pnas.1322923111 24550284 PMC3932880

[B93] Paesen GC Happ GM The B proteins secreted by the tubular accessory sex glands of the male mealworm beetle, Tenebrio molitor, have sequence similarity to moth pheromone-binding proteins Insect Biochem Mol Biol 1995 25 401 8 10.1016/0965-1748(94)00085-V 7773257

[B94] Thymianou S Mavroidis M Kokolakis G Komitopoulou K Zacharopoulou A Mintzas AC Cloning and characterization of a cDNA encoding a male-specific serum protein of the Mediterranean fruit fly, Ceratitis capitata, with sequence similarity to odorant-binding proteins Insect Mol Biol 1998 7 345 53 10.1046/j.1365-2583.1998.740345.x 9723872

[B95] Paiva-Silva GO Sorgine MHF Benedetti CE Meneghini R Almeida IC Machado EA Dansa-Petretski M Yepiz-Plascencia G Law JH Oliveira PL Masuda H On the biosynthesis of Rhodnius prolixus heme-binding protein Insect Biochem Mol Biol 2002 32 1533 41 10.1016/S0965-1748(02)00074-7 12530221

[B96] Forêt S Maleszka R Function and evolution of a gene family encoding odorant binding-like proteins in a social insect, the honey bee (Apis mellifera) Genome Res 2006 16 1404 13 10.1101/gr.5075706 17065610 PMC1626642

[B97] Vogt RG Prestwich GD Lerner MR Odorant-binding-protein subfamilies associate with distinct classes of olfactory receptor neurons in insects J Neurobiol 1991 22 74 84 10.1002/neu.480220108 2010751

[B98] Arya GH Weber AL Wang P Magwire MM Negron YL Mackay TF Anholt RR Natural variation, functional pleiotropy and transcriptional contexts of odorant binding protein genes in Drosophila melanogaster Genetics 2010 186 1475 85 10.1534/genetics.110.123166 20870963 PMC2998325

[B99] Zhou S Stone EA Mackay TF Anholt RR Plasticity of the chemoreceptor repertoire in Drosophila melanogaster PLoS Genet 2009 5 e1000681 10.1371/journal.pgen.1000681 19816562 PMC2750752

[B100] Kodrík D Filippov VA Sehnal F Filippova MA Sericotropin: an insect neurohormonal factor affecting RNA transcription Netherlands J Zool 1995

[B101] Nagnan-Le Meillour P Cain AH Jacquin-Joly E François MC Ramachandran S Maida R Steinbrecht RA Chemosensory proteins from the proboscis of mamestra brassicae Chem Senses 2000 25 541 53 10.1093/chemse/25.5.541 11015326

[B102] Jacquin-Joly E Vogt RG François MC Nagnan-Le Meillour P Functional and expression pattern analysis of chemosensory proteins expressed in antennae and pheromonal gland of Mamestra brassicae Chem Senses 2001 26 833 44 10.1093/chemse/26.7.833 11555479

[B103] Wanner KW Willis LG Theilmann DA Isman MB Feng Q Plettner E Analysis of the insect os-d-like gene family J Chem Ecol 2004 30 889 911 15274438 10.1023/b:joec.0000028457.51147.d4

[B104] Walensky LD Ruat M Bakin RE Blackshaw S Ronnett G V Snyder SH Two novel odorant receptor families expressed in spermatids undergo 5'-splicing J Biol Chem 1998 273 9378 87 10.1074/jbc.273.16.9378 9545261

[B105] Suzuki N Garbers DL Stimulation of sperm respiration rates by speract and resact at alkaline extracellular pH Biol Reprod 1984 30 1167 74 10.1095/biolreprod30.5.1167 6547354

[B106] Parmentier M Libert F Schurmans S Schiffmann S Lefort A Eggerickx D Ledent C Mollereau C Gérard C Perret J Expression of members of the putative olfactory receptor gene family in mammalian germ cells Nature 1992 355 453 5 10.1038/355453a0 1370859

[B107] Ogaugwu CE Schetelig MF Wimmer EA Transgenic sexing system for Ceratitis capitata (Diptera: Tephritidae) based on female-specific embryonic lethality Insect Biochem Mol Biol 2013 43 1 8 10.1016/j.ibmb.2012.10.010 23137881

[B108] Hanife G Embryonic development of the olive fruit fly, Bactrocera oleae Rossi ( Diptera: Tephritidae ), in vivo Turkish J Zool 2014

[B109] Schweisguth F Lepesant JA Vincent A The serendipity alpha gene encodes a membrane-associated protein required for the cellularization of the Drosophila embryo Genes Dev 1990 4 922 31 10.1101/gad.4.6.922 2166703

[B110] Ibnsouda S Schweisguth F de Billy G Vincent A Relationship between expression of serendipity alpha and cellularisation of the Drosophila embryo as revealed by interspecific transformation Development 1993 119 471 83 8287797 10.1242/dev.119.2.471

[B111] Schmid KJ Tautz D A screen for fast evolving genes from Drosophila Proc Natl Acad Sci USA 1997 94 9746 50 10.1073/pnas.94.18.9746 9275195 PMC23261

[B112] Holt RA Subramanian GM Halpern A Sutton GG Charlab R Nusskern DR Wincker P Clark AG Ribeiro JM Wides R Salzberg SL Loftus B Yandell M Majoros WH Rusch DB Lai Z Kraft CL Abril JF Anthouard V Arensburger P Atkinson PW Baden H de Berardinis V Baldwin D Benes V Biedler J Blass C Bolanos R Boscus D Barnstead M et al The genome sequence of the malaria mosquito Anopheles gambiae Science 2002 298 129 49 10.1126/science.1076181 12364791

[B113] Zou Z Lopez DL Kanost MR Evans JD Jiang H Comparative analysis of serine protease-related genes in the honey bee genome: possible involvement in embryonic development and innate immunity Insect Mol Biol 2006 15 603 14 10.1111/j.1365-2583.2006.00684.x 17069636 PMC1761132

[B114] Haugen M Flannery E Tomchaney M Mori A Behura SK Severson DW Duman-Scheel M Semaphorin-1a is required for Aedes aegypti embryonic nerve cord development PLoS One 2011 6 e21694 10.1371/journal.pone.0021694 21738767 PMC3124551

[B115] Schetelig MF Horn C Handler AM Wimmer EA MJB Vreysen, AS Robinson J Hendrichs Development of an Embryonic Lethality System in Mediterranean Fruit Fly Ceratitis capitata Area-Wide Control Insect Pests 2007 85 93

[B116] Tadros W Lipshitz HD The maternal-to-zygotic transition: a play in two acts Development 2009 136 3033 42 10.1242/dev.033183 19700615

[B117] Schetelig MF Caceres C Zacharopoulou A Franz G Wimmer EA Conditional embryonic lethality to improve the sterile insect technique in Ceratitis capitata (Diptera: Tephritidae) BMC Biol 2009 7 4 10.1186/1741-7007-7-4 19173707 PMC2662800

[B118] Gabrieli P Gomulski LM Bonomi A Siciliano P Scolari F Franz G Jessup A Malacrida AR Gasperi G Interchromosomal duplications on the Bactrocera oleae Y chromosome imply a distinct evolutionary origin of the sex chromosomes compared to Drosophila PLoS One 2011 6 e17747 10.1371/journal.pone.0017747 21408187 PMC3049792

[B119] Bilak A Su TT Regulation of Drosophila melanogaster pro-apoptotic gene hid Apoptosis 2009 14 943 9 10.1007/s10495-009-0374-2 19554451 PMC3373429

[B120] Kornbluth S White K Apoptosis in Drosophila: neither fish nor fowl (nor man, nor worm) J Cell Sci 2005 118 Pt 9 1779 87 15860727 10.1242/jcs.02377

[B121] Hay BA Guo M Caspase-dependent cell death in Drosophila Annu Rev Cell Dev Biol 2006 22 623 50 10.1146/annurev.cellbio.21.012804.093845 16842034

[B122] Steller H Regulation of apoptosis in Drosophila Cell Death Differ 2008 15 1132 8 10.1038/cdd.2008.50 18437164

[B123] Horn C Wimmer EA A transgene-based, embryo-specific lethality system for insect pest management Nat Biotechnol 2003 21 64 70 12483222 10.1038/nbt769

[B124] Schetelig MF Nirmala X Handler AM Pro-apoptotic cell death genes, hid and reaper, from the tephritid pest species, Anastrepha suspensa Apoptosis 2011 16 759 68 10.1007/s10495-011-0610-4 21630017

[B125] Grether ME Abrams JM Agapite J White K Steller H The head involution defective gene of Drosophila melanogaster functions in programmed cell death Genes Dev 1995 9 1694 708 10.1101/gad.9.14.1694 7622034

[B126] Abrams JM White K Fessler LI Steller H Programmed cell death during Drosophila embryogenesis Development 1993 117 29 43 8223253 10.1242/dev.117.1.29

[B127] M.E Economopoulos A Tsitsipis J The importance of conditions during the adult stage in evaluating an artificial food for larvae of Dacus oleae (Gmel.) (Diptera, Tephritidae) Z Angew Entomol 1967 59 127 130

[B128] Tsitsipis J Development of a caging and egging system for mass rearing the olive fruit fly, Dacus oleae (Gmel.) (Diptera, Tephritidae) Ann Zool Ecol Anim 1977 9 133 139

[B129] Tsitsipis JA Kontos A Improved solid adult diet for the olive fruit fly Dacus oleae Entomol Hell 1983 1 24 29

[B130] Camacho C Coulouris G Avagyan V Ma N Papadopoulos J Bealer K Madden TL BLAST+: architecture and applications BMC Bioinformatics 2009 10 421 10.1186/1471-2105-10-421 20003500 PMC2803857

[B131] Trapnell C Pachter L Salzberg SL TopHat: discovering splice junctions with RNA-Seq Bioinformatics 2009 25 1105 11 10.1093/bioinformatics/btp120 19289445 PMC2672628

[B132] Blüthgen N Brand K Cajavec B Swat M Herzel H Beule D Biological profiling of gene groups utilizing Gene Ontology Genome Inform 2005 16 106 15 16362912

