# Supplementary material for: The molecular biology of the olive fly comes of age
Source: BMC Genet. 2014 Dec 1;15(Suppl 2):S8. doi: 10.1186/1471-2156-15-S2-S8 (PMC4255830; doi:10.1186/1471-2156-15-S2-S8)
Supplement: Additional File 1 [file 1471-2156-15-S2-S8-S1.docx]

**Table S1**

| **Top 40 female genes of *B. oleae*** | | | |
| --- | --- | --- | --- |
| **Gene ID** | **Gene name [Species]** | **q Value** | **Log2**  **(fold change)** |
| XP_002010117 | GI15747 [Drosophila mojavensis] | 4,73E-009 | -8,21227 |
| XP_001993685 | GH21097 [Drosophila grimshawi] | 0,0024075 | -9,94448 |
| AEJ88361 | peroxidase isoform A [Bactrocera dorsalis] | 0,0024075 | -9,72142 |
| XP_001661917 | venom allergen [Aedes aegypti] | 0,0024075 | -9,69208 |
| AAQ17521 | glucose dehydrogenase [Drosophila erecta] | 0,0024075 | -9,358 |
| AAY98012 | serine protease Ssp3-2 precursor [Stomoxys calcitrans] | 0,0024075 | -9,104 |
| EHK24630 | hypothetical protein TRIVIDRAFT_71933 [Trichoderma virens Gv29-8] | 0,00248048 | -9,55711 |
| XP_001358555 | GA20090 [Drosophila pseudoobscura pseudoobscura] | 0,00248048 | -9,14296 |
| XP_002059659 | GJ14891 [Drosophila virilis] | 0,00248048 | -8,9536 |
| XP_002134111 | GA29233 [Drosophila pseudoobscura pseudoobscura] | 0,00362803 | -8,45892 |
| XP_002082774 | GD11758 [Drosophila simulans] | 0,00362803 | -8,42249 |
| EGX96098 | tRNA ligase [Cordyceps militaris CM01] | 0,00367952 | -8,48353 |
| XP_001986176 | GH21214 [Drosophila grimshawi] | 0,00393291 | -8,24049 |
| XP_002006001 | GI18785 [Drosophila mojavensis] | 0,00404218 | -9,42795 |
| AAX13070 | glucose dehydrogenase [Drosophila miranda] | 0,00404218 | -8,76807 |
| EFN79524 | hypothetical protein EAI_06736 [Harpegnathos saltator] | 0,00404218 | -8,72969 |
| ADQ64521 | hypothetical protein [Bactrocera oleae] | 0,00404218 | -8,64223 |
| ADD18879 | salivary antigen 5 precursor [Glossina morsitans morsitans] | 0,00404218 | -8,25497 |
| EFN71731 | hypothetical protein EAG_11590 [Camponotus floridanus] | 0,00404218 | -8,25038 |
| AAR10045 | similar to Drosophila melanogaster Ag5r, partial [Drosophila yakuba] | 0,00404218 | -8,16695 |
| XP_001985929 | GH20821 [Drosophila grimshawi] | 0,00404218 | -8,03683 |
| XP_002071447 | GK25805 [Drosophila willistoni] | 0,00404218 | -7,97015 |
| AAM00373 | vitellogenin 2 precursor [Bactrocera dorsalis] | 0,00404218 | -7,96415 |
| XP_002053787 | GJ23153 [Drosophila virilis] | 0,00404218 | -7,93173 |
| XP_001959800 | GF11856 [Drosophila ananassae] | 0,00404218 | -7,86093 |
| YP_003427454 | polycyclic aromatic hydrocarbon dioxygenase large subunit  [Bacillus pseudofirmus OF4] | 0,00416687 | -8,35647 |
| XP_002017169 | GL22161 [Drosophila persimilis] | 0,00451292 | -9,19682 |
| AAB87896 | glucose dehydrogenase [Drosophila subobscura] | 0,00451292 | -7,81215 |
| AAB61390 | transposase [Hydra vulgaris] | 0,00458371 | -8,89647 |
| XP_001354610 | GA18700 [Drosophila pseudoobscura pseudoobscura] | 0,00458371 | -8,39457 |
| NP_989320 | serine protease ami precursor [Xenopus (Silurana) tropicalis] | 0,00458371 | -8,13941 |
| XP_002013949 | GL24419 [Drosophila persimilis] | 0,00458371 | -8,10711 |
| ZP_14656410 | PF12650 domain protein [Capnocytophaga sp. oral taxon 335 str. F0486] | 0,00458371 | -7,81299 |
| AAM00372 | vitellogenin 1 precursor [Bactrocera dorsalis] | 0,00458371 | -7,77836 |
| AAK51353 | anterior fat body protein [Calliphora vicina] | 0,00458371 | -7,75168 |
| XP_003458838 | PREDICTED: trypsin-1-like [Oreochromis niloticus] | 0,00458371 | -7,62035 |
| NP_731781 | CG8630 [Drosophila melanogaster] | 0,00458371 | -7,55072 |
| XP_001374677 | PREDICTED: 60S acidic ribosomal protein P2-like  [Monodelphis domestica] | 0,00458371 | -7,55072 |
| XP_001843958 | deoxyribonuclease I [Culex quinquefasciatus] | 0,00458371 | -7,533 |
| NP_995924 | CG9826, isoform B [Drosophila melanogaster] | 0,00458371 | -7,50848 |

| **Top 40 male genes of *B. oleae*** | | | |
| --- | --- | --- | --- |
| **Gene ID** | **Gene name [Species]** | **q Value** | **Log2**  **(fold change)** |
| XP_002008256 | GI13389 [Drosophila mojavensis] | 0,0025 | 8,87716 |
| XP_002160969 | PREDICTED: similar to GF20795 [Hydra magnipapillata] | 0,0025 | 9,28259 |
| XP_001357953 | GA14948 [Drosophila pseudoobscura pseudoobscura] | 0,003628 | 8,51638 |
| XP_002064007 | GK15975 [Drosophila willistoni] | 0,003628 | 8,78001 |
| EKE06479 | NUDIX hydrolase [uncultured bacterium] | 0,00368 | 8,833 |
| ACE79730 | coiled-coil Y protein [Drosophila virilis] | 0,00368 | 8,84812 |
| XP_002038214 | GM17875 [Drosophila sechellia] | 0,004042 | 7,93615 |
| XP_002080489 | GD10219 [Drosophila simulans] | 0,004042 | 7,96926 |
| XP_002078278 | GD22629 [Drosophila simulans] | 0,004042 | 8,02622 |
| XP_001980969 | GG10368 [Drosophila erecta] | 0,004042 | 8,1338 |
| YP_001943997 | chromosome segregation protein SMC [Chlorobium limicola DSM 245] | 0,004042 | 8,45646 |
| ZP_10867140 | flagellar export protein FliJ [Paenibacillus alvei DSM 29] | 0,004042 | 8,64318 |
| XP_001351577 | chromosome assembly factor 1, CAF-1 [Plasmodium falciparum 3D7] | 0,004167 | 7,76928 |
| XP_002102115 | GD19672 [Drosophila simulans] | 0,004167 | 7,79838 |
| XP_002056731 | GJ11096 [Drosophila virilis] | 0,004167 | 7,87051 |
| XP_002059967 | GJ14950 [Drosophila virilis] | 0,004167 | 7,91428 |
| XP_001957893 | GF23790 [Drosophila ananassae] | 0,004167 | 8,33505 |
| EFZ14132 | hypothetical protein SINV_09504 [Solenopsis invicta] | 0,004167 | 8,33934 |
| XP_001970189 | GG10492 [Drosophila erecta] | 0,004444 | 7,82385 |
| XP_001965705 | GF22311 [Drosophila ananassae] | 0,004513 | 7,79916 |
| XP_001846016 | conserved hypothetical protein [Culex quinquefasciatus] | 0,004513 | 7,93567 |
| EFR19322 | hypothetical protein AND_22695 [Anopheles darlingi] | 0,00456 | 8,20952 |
| XP_002056267 | GJ10850 [Drosophila virilis] | 0,004584 | 7,35552 |
| NP_001097458 | CG34453 [Drosophila melanogaster] | 0,004584 | 7,3676 |
| XP_002021125 | GL24992 [Drosophila persimilis] | 0,004584 | 7,37597 |
| XP_001956989 | GF24290 [Drosophila ananassae] | 0,004584 | 7,38982 |
| YP_006440826 | HAD-superfamily hydrolase, subfamily IA, variant 1  [Turneriella parva DSM 21527] | 0,004584 | 7,39463 |
| XP_002053337 | GJ23394 [Drosophila virilis] | 0,004584 | 7,45251 |
| XP_002091757 | GE12070 [Drosophila yakuba] | 0,004584 | 7,46368 |
| XP_001967351 | GF13897 [Drosophila ananassae] | 0,004584 | 7,46891 |
| XP_002063726 | GK15752 [Drosophila willistoni] | 0,004584 | 7,48448 |
| NP_730300 | CG32181 [Drosophila melanogaster] | 0,004584 | 7,4954 |
| XP_002033365 | GM20454 [Drosophila sechellia] | 0,004584 | 7,52016 |
| XP_001843421 | paramyosin [Culex quinquefasciatus] | 0,004584 | 7,54822 |
| XP_002366878 | hypothetical protein, conserved [Toxoplasma gondii ME49] | 0,004584 | 7,60273 |
| XP_002014988 | GL19472 [Drosophila persimilis] | 0,004584 | 7,61693 |
| XP_001956365 | GF25173 [Drosophila ananassae] | 0,004584 | 7,65526 |
| NP_998476 | purine nucleoside phosphorylase 5a [Danio rerio] | 0,004584 | 7,79573 |
| XP_001962841 | GF14227 [Drosophila ananassae] | 0,004584 | 7,88525 |
| XP_001953934 | GF18016 [Drosophila ananassae] | 0,004584 | 7,89693 |
