# Supplementary material for: The molecular biology of the olive fly comes of age
Source: BMC Genet. 2014 Dec 1;15(Suppl 2):S8. doi: 10.1186/1471-2156-15-S2-S8 (PMC4255830; doi:10.1186/1471-2156-15-S2-S8)
Supplement: Additional File 1 [file 1471-2156-15-S2-S8-S2.docx]

| **Table S2. Primer sequences used for qRT-PCR** | | | |
| --- | --- | --- | --- |
|  |  |  |  |
| Gene name | Forward primer (5’-3’) | Reverse Primer (5’-3’) | Amplicon size (bp) |
| **Sex-specific genes** | | | |
| *kl3* | CGC TCT TGT ACA ACT GTA TG | TAC CTA TGG CAA TAT GTC AC | 105 |
| *kl5* | CAA TTG CGA TGT GAC TAA G | CCT TAA GAA AAG CAT CAG C | 137 |
| *ory* | CGT CTT CTT GTA TTT CTT GG | GAA CTA CGT AAG GAT CAA CG | 111 |
| *gas8* | AGA TGG AGC GTG AAC GTG AAG | CGT GAT CCT TGT GAC GAA CC | 126 |
| *lobo* | CTG CAA TGG TAG TAT CTG G | GAT CTC TTT GAT TTG GTG TG | 89 |
| *fem-1* | ACA AGA GAA GAC TGC CCT AC | TGA CTT GCT CCT TTA ATG C | 146 |
| *ix* | CTG TCG CCA TTA CGT GAG TC | CGT CGC GTT TAA GAT TAT CTG C | 94 |
| *pbl* | TAC CGA GTC CAA CTA TGT G | TAT TGA GTA AAG CGT CGT TG | 98 |
| *hcf* | CAA CGT GTT AAT CTT GAG C | TCA CTC CAA TCA CTA GCA C | 83 |
| *pcp* | GTG AGG TGG CCT TCT TCA GG | GCA GGT GCA TCA ATT GTT AGT C | 124 |
| *sox* | AGC ACA GCG GCT GAA TTT TG | AGT CAC AGT CCG AGC GAT TC | 92 |
| **Olfactory genes** | | | |
| *Obp3* | ACA GAG GAG GCA ATT AAG | ATC ACC GTT ATC ATC CAC | 119 |
| *Obp20* | AAG GAG GAT TAT CGC AAC | AAT TAG AAG GGC ATA AGA CG | 90 |
| *Obp1* | AAG GCG AAT ACG GAA GTG C | CTG ACC CAC CTG ACT GTT TAG | 123 |
| *ChemR* | CCT GGA CGA GGT TTT GAG C | TTG ATA TAG CGT CGG GCA GTA TC | 122 |
| *Or10* | AGC TCT TCA ATT TCT TGT TGC TGT | CAT CGC TTG AGC CAT TCT TCG | 60 |
| **Embryonic development genes** | | | |
| *sry-a* | GTG CGG TAA GCG ATT CCA | CGA CAC TGC CTG AAC TGA | 118 |
| *hid* | GAT TGC ATC GTA GCC TCT CA | AAA CGG TCA TCG AAC TCA TC | 120 |
| **Housekeeping genes** | | | |
| *Rpl19* | CTT CAC GTA CTT TAT GCC TTC | GCA AGG GTA ATG TGT TCA A | 126 |
| *14-3-3zeta* | GTC TTC TCG ACA AAC ACC | CCA CCT CAG CTA AAT ACC | 102 |
| *actin3* | GGT CGG TAT GGG ACA GAA GG | CTC ACG ATT GGC TTT TGG AT | 220 |
| *α-tubulin* | TAC ATG GCC TGT TGT ATG | GCT TGG TCT TGA TGG TAG | 82 |

* All the primers were constructed based on the RNA-seq data of the *B. oleae* transcriptome. Only the set of primers for the gene actin3 was used from Shen et al 2010.
